# Optogenetic Control of Dopamine Receptor 2 Reveals a Novel Aspect of Dopaminergic Neurotransmission in Motor Function

**DOI:** 10.1523/JNEUROSCI.1473-24.2024

**Published:** 2024-11-19

**Authors:** Hyunbin Kim, Geunhong Park, Hyo Geun Shin, Duwan Kwon, Heejung Kim, In-Yeop Baek, Min-Ho Nam, Il-Joo Cho, Jeongjin Kim, Jihye Seong

**Affiliations:** ^1^Brain Science Institute, Korea Institute of Science and Technology, Seoul 02792, Republic of Korea; ^2^Department of Pharmacology, Seoul National University College of Medicine, Seoul 03080, Republic of Korea; ^3^Neuroscience Research Institute, Medical Research Institute, Seoul National University College of Medicine, Seoul 03080, Republic of Korea; ^4^School of Electronic and Electrical Engineering, Kyungpook National University, Daegu 41566, Republic of Korea; ^5^Department of Biomedical Sciences, Seoul National University College of Medicine, Seoul 03080, Republic of Korea; ^6^Department of KHU-KIST Convergence Science and Technology, Kyung Hee University, Seoul 02447, Republic of Korea; ^7^Departments of Convergence Medicine, Korea University, Seoul 02841, Republic of Korea; ^8^Anatomy, College of Medicine, Korea University, Seoul 02841, Republic of Korea; ^9^Division of Bio-Medical Science & Technology, University of Science and Technology, Daejeon 34113, Republic of Korea; ^10^Interdisciplinary Program in Neuroscience, Seoul National University, Seoul 08826, Republic of Korea

**Keywords:** dopamine receptor 2, lateral globus pallidus (LGP), motor function, OptoDRD2, optogenetics

## Abstract

Dopaminergic neurotransmission plays a crucial role in motor function through the coordination of dopamine receptor (DRD) subtypes, such as DRD1 and DRD2, thus the functional imbalance of these receptors can lead to Parkinson's disease. However, due to the complexity of dopaminergic circuits in the brain, it is limited to investigating the individual functions of each DRD subtype in specific brain regions. Here, we developed a light-responsive chimeric DRD2, OptoDRD2, which can selectively activate DRD2-like signaling pathways with spatiotemporal resolution. OptoDRD2 was designed to include the light-sensitive component of rhodopsin and the intracellular signaling domain of DRD2. Upon illumination with blue light, OptoDRD2 triggered DRD2-like signaling pathways, such as Gαi/o subtype recruitment, a decrease in cAMP levels, and ERK phosphorylation. To explore unknown roles of DRD2 in glutamatergic cell populations of basal ganglia circuitry, OptoDRD2 was genetically expressed in excitatory neurons in lateral globus pallidus (LGP) of the male mouse brain. The optogenetic stimulation of OptoDRD2 in the LGP region affected a wide range of locomotion-related parameters, such as increased frequency of movement and decreased immobility time, resulting in the facilitation of motor function of living male mice. Therefore, our findings indicate a potentially novel role for DRD2 in the excitatory neurons of the LGP region, suggesting that OptoDRD2 can be a valuable tool enabling the investigation of unknown roles of DRD2 at specific cell types or brain regions.

## Significance Statement

We developed a light-responsive chimeric dopamine receptor type 2, OptoDRD2, by combining the blue light-sensing part of rhodopsin and intracellular functional regions of DRD2. OptoDRD2 can selectively trigger DRD2-like downstream signaling pathways upon illumination of blue light. To explore the unknown roles of DRD2 in glutamatergic cell populations of basal ganglia circuitry, OptoDRD2 was genetically expressed in excitatory neurons at lateral globus pallidus (LGP) in the mouse brain. Optogenetic stimulation of OptoDRD2 in living mice suggested a potential novel function of DRD2 in the LGP that enhances motor outputs. Therefore, OptoDRD2 enabled the precise control of DRD2-like signaling in specific cell types and brain regions, allowing the exploration of potential novel DRD2 functions in living mice.

## Introduction

Dopaminergic neurotransmission plays important roles in the regulation of reward process, motivation, moods, and locomotion (Wise, 2004; Beaulieu and Gainetdinov, 2011). During dopaminergic neurotransmission, dopamine is released from presynaptic neurons to the synaptic cleft, and the released dopamine binds to its receptors in the postsynaptic neurons initiating the dopaminergic signaling pathways (Tritsch and Sabatini, 2012). Dopamine receptors (DRDs), and many other neurotransmitter receptors, belong to the superfamily of G-protein–coupled receptors (GPCRs; Missale et al., 1998; Beaulieu et al., 2015). Upon the binding of extracellular ligands such as dopamine, the activated GPCRs change their conformation particularly in the intracellular loop (ICL) regions (Zhuang et al., 2021). The corresponding G-proteins are then recruited to the intracellular loop regions of the activated GPCRs, initiating the downstream signaling, for example, dopaminergic pathways (Pierce et al., 2002; Thal et al., 2018). Therefore, the activation of dopamine receptors is a crucial step for dopaminergic neurotransmission.

There are five subtypes of dopamine receptors, DRD1–DRD5, which display different expression levels and distribution in the brain (Beaulieu et al., 2015). These DRD subtypes also exhibit different binding affinities to dopamine. For example, the dopamine binding affinity of DRD1 and DRD2, two major DRD subtypes for motor function, is suggested to be micromolar and nanomolar range, respectively, suggesting differential activation status of DRD1 and DRD2 in response to various dopamine concentrations (Knight et al., 2003; Marcellino et al., 2012). More interestingly, DRD1 and DRD2 recruit different Gα proteins, i.e., Gαs and Gαi, which mediate opposite effects on the cAMP levels and thus the related downstream signaling pathways (Neve et al., 2004; Loewke et al., 2021; H. Kim et al., 2022). Therefore, the activation of individual DRDs should be investigated to fully understand complex dopaminergic neurotransmission.

We previously developed multicolor fluorescent biosensors that detect individual activities of DRD1 or DRD2 during dopaminergic neurotransmission (H. Kim et al., 2022). In addition to DRD biosensors, optogenetic tools capable of selectively activating specific DRDs would be beneficial for identifying the exact functions of DRD1 or DRD2 in the dopaminergic circuits of the brain. For the optical activation of GPCRs, the concept of OptoXRs was previously introduced with OptoB2AR, a chimeric GPCR composed of the light-sensing part of bovine rhodopsin and the functional domain of B2AR (Airan et al., 2009). Upon illumination of blue light, OptoB2AR changes its conformation and recruits Gαs protein, which then initiates the B2AR-specific signaling events. Following this strategy, OptoDRD1 was developed to investigate the direct role of DRD1 in medium spiny neurons for the reward function at the VTA of the mouse brain (Gunaydin et al., 2014).

In this study, we aimed to develop OptoDRD2 to selectively control the DRD2-like downstream signaling with high spatiotemporal resolution. OptoDRD2 was designed by combining the extracellular and transmembrane domains of bovine rhodopsin and the intracellular domain of DRD2. Upon illumination with blue light, OptoDRD2 induced the DRD2-like signaling pathways, such as Gαi/o subtype recruitment, the reduction in cAMP, and the phosphorylation of ERK (Nishi et al., 2000). Importantly, OptoDRD2 induced distinct signaling responses compared with OptoDRD1, suggesting that we can selectively control the DRD1- and DRD2-like signaling pathways by light. To further explore the function of DRD2 in glutamatergic cell populations of basal ganglia circuitry (Dong et al., 2021), OptoDRD2 was genetically expressed in the excitatory neurons at the lateral globus pallidus (LGP) of the mouse brain. The optogenetic stimulation on the OptoDRD2 in these excitatory LGP neurons can affect a wide range of movement parameters such as movement frequency and time spent in mobile states. Therefore, we have developed a novel optogenetic strategy to selectively activate DRD2-like signaling pathways and successfully applied OptoDRD2 to mice to explore the potential novel DRD2 functions in the specific brain region.

## Materials and Methods

### Plasmids

**Figure 1.**
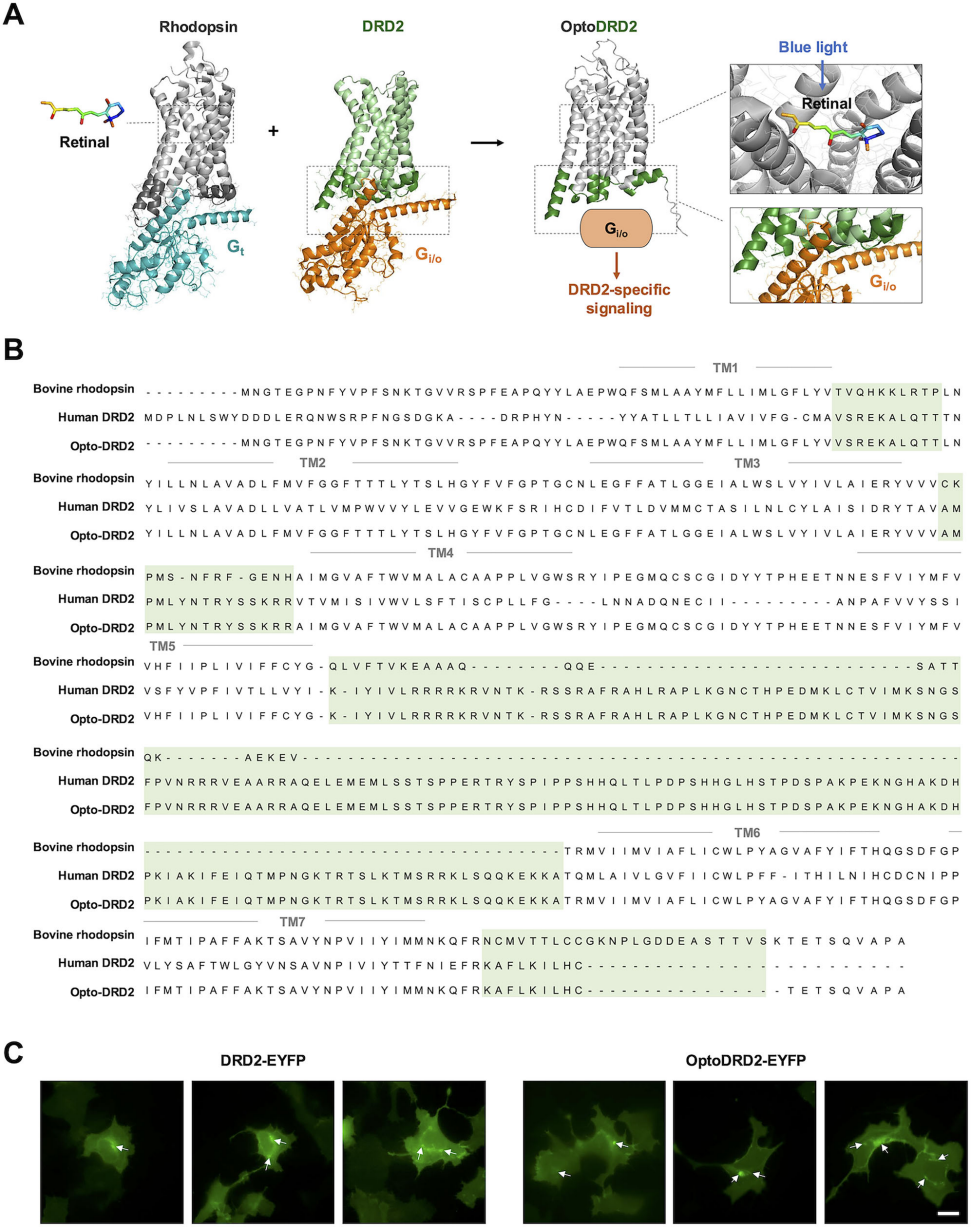
Design of OptoDRD2. ***A***, Design strategy for OptoDRD2. Based on the structures of the bovine rhodopsin-Gαt complex (modified from PDB 6OYA) and DRD2-Gαi/o complex (modified from PDB 7JVR), we expect that OptoDRD2 can mediate the DRD2-like signaling pathways in response to blue light. The structure of OptoDRD2 is predicted from AlphaFold. Gray residues are from rhodopsin and green residues are from DRD2. ***B***, Sequence alignment of bovine rhodopsin, human DRD2, and OptoDRD2. The green highlights the intracellular regions of DRD2 which are used in OptoDRD2. TM, transmembrane domains. ***C***, Representative images of DRD2-EYFP (left) and OptoDRD2-EYFP (right) in HEK293A cells. The white arrows indicate the distinctive intracellular distribution. Scale bar, 20 μm.

Human GPCR sequences in pcDNA5 vectors, OptoDRD1, KORD, and OptoMOR constructs were achieved from Addgene. For the design of OptoDRD2, the sequences of bovine rhodopsin and the one of DRD2 were aligned (Fig. 1). After extracellular and transmembrane domains of rhodopsin and intracellular loops of human DRD2 were amplified by PCR, they were fused and inserted into the pcDNA5 vector by In-Fusion (Clontech). To check the expression of OptoDRD2, EYFP was amplified by PCR and fused to the C terminal of OptoDRD2. For in vivo application, OptoDRD2-EYFP was subcloned into a pAAV-Ef1a-DIO vector, and viruses were produced in the Korea Institute of Science and Technology (KIST) virus facility. pink-Flamindo was obtained from Addgene, and pGlo-22F was obtained from Promega.

### Cell culture and reagents

The human embryonic kidney 293A (HEK293A) cell line was maintained in DMEM supplemented with 10% fetal bovine serum (Hyclone), 1 unit per ml penicillin, 100 μg per ml streptomycin, and 100 μM MEM nonessential amino acid solution (Gibco). Cell culture reagents were purchased from Hyclone. Cells were cultured in a humidified 95% air and 5% CO_2_ incubator at 37°C. Lipofectamine 2000 Reagent (Invitrogen) was used for the transfection of plasmids according to the manufacturer's protocol. For the live-cell fluorescence imaging, the transfected cells were seeded on cover glass-bottom dishes (SPL) at a density of 1 × 10^5^ cells for 24 h. Quinpirole and 9-*cis*-retinal were purchased from Sigma-Aldrich, and forskolin (FK) was purchased from Tocris Bioscience. D-Luciferin sodium salt for the cAMP assay was purchased from GoldBio.

### Light emitting diode (LED) device for cell illumination experiment

For the illumination of OptoDRD2 while live-cell imaging with an inverted fluorescence microscope, we constructed a customized LED which can illuminate the cells from the top side of the dish. The LED device consists of 25 blue LEDs, a potentiometer to adjust light intensity, and a wireless controller comprised of a microcontroller and a Bluetooth module for on/off control of the LEDs (Extended Data Fig. 2-1). Specifically, the high-intensity LEDs with 1.6 mm × 1.6 mm are evenly spaced at 7 mm intervals, enabling uniform light illumination to the area with 32.5 mm × 32.5 mm. The light intensity is adjustable from 0.23 to 11 mW by rotating the potentiometer (Extended Data Fig. 2-2). The 25 LEDs are wirelessly controlled by the integrated wireless controller. Also, the duty cycles of the LEDs are controlled by programs downloaded in the microcontroller for up to eight conditions.

### Live-cell imaging and image acquisition

Live-cell imaging was performed in a humidified 95% air, 5% CO_2_, and 37°C temperature-controlled chamber (Live Cell Instrument). The HEK293A cells expressing pink-Flamindo together with OptoDRD1 or OptoDRD2 were prepared on the cover glass-bottom dishes coated with 10 μg/ml of fibronectin (Invitrogen). Before the light stimulation on OptoDRD2, the cells were incubated with 9-*cis*-retinal (1 μM) for 24 h. Images were collected by a Nikon Ti-E inverted microscope and a cooled charge-coupled device camera using NIS software (Nikon).

For measuring the cAMP levels, red fluorescent signals of pink-Flamindo were collected every minute using a 562DF40 excitation filter, a 593DRLP, and a 641DF75 emission filter, with an exposure time of 50 ms under a 100× objective. For confirming OptoDRD2-EYFP expression, yellow fluorescent signals were collected using a 482DF35 excitation filter, a 506DRLP dichroic mirror, and a 536DF40 emission filter, with an exposure time of 50 ms. The fluorescence intensity of nontransfected cells was quantified as the background signal and subtracted from the fluorescence signals from transfected cells. The pixel-by-pixel fluorescent intensity images were calculated based on the background-subtracted fluorescence intensity images of pink-Flamindo by the NIS program to allow the quantification and statistical analysis.

### Glosensor cAMP assay

For the GloSensor cAMP assay (Fan et al., 2008), pGlo-22F and GPCR plasmids were cotransfected in HEK293A cells for 24–36 h. The transfected cells were harvested and seeded on 96-well plates at a density of 2 × 10^4^ cells for 24 h. Before the assay, we confirmed 60–80% transfection efficiency of GPCR-EYFP in the cells. On the day of the assay, the culture media was removed and replaced by HBSS buffer (pH 7.4) with 20 mM HEPES and D-luciferin sodium salt (GoldBio, 150 μg/ml). Then 9-*cis*-retinal (1 μM) was added to the wells and incubated for 30 min at 37°C. After light illumination with or without the treatment with forskolin (10 μM), the luminescence signals of the GloSensor were measured every 2 min using a Synergy H1 microplate reader (BioTek Instruments).

### Western blotting

HEK293A cells were harvested on the ice with 1× lysis buffer (Cell Signaling Technology, catalog #9803) containing 1 mM PMSF, mixed with 5× sample buffer [250 mM Tris-HCl (pH 6.8), 0.5 M DTT, 10% SDS, 50% glycerol, 0.2% bromophenol blue], and boiled at 100°C for 5 min. The prepared samples (15 μg) were loaded on the 10 or 12% SDS–PAGE gels. The separated proteins were transferred to the nitrocellulose membrane (Thermo Fisher Scientific), and the membranes were blocked with 5% skim milk in TBST (DIFCO) for 1 h at room temperature (RT). After blocking, the membranes were incubated with anti-pDARPP-32 (Thr34; 5 μg/ml, Cell Signaling Technology, catalog #12438), anti-pERK1/2 (1 μg/ml, Cell Signaling Technology, catalog #9101), anti-ERK1/2 (1 μg/ml, Cell Signaling Technology, catalog #4695), or anti-DARPP-32 (5 μg/ml, Santa Cruz Biotechnology, catalog #sc-271111) antibodies at 4°C for overnight. The membranes were washed with TBST buffer and then incubated with HRP-conjugated rabbit secondary antibody (1 μg/ml, VectorLabs, catalog #PI-1000) or HRP-conjugated mouse secondary antibody (1 μg/ml, Santa Cruz Biotechnology, catalog #sc-2005) for 2 h at RT. Chemiluminescence signals were developed by ECL solution (pico or femto, Thermo Fisher Scientific) and detected by Amersham ImageQuant 800 (GE HealthCare).

### Gα protein coupling assay

HEK293A cells were seeded in six-well plates (Corning) at 4 × 10^5^ cells per well. Plasmids encoding GPCR of interest (DRD2, OptoDRD2, KORD, OptoMOR), Gαi/o subtypes tagged with Rluc8 (i1, i2, i3, oA, oB), Gβ3, and mCitrine-Gγ9 were cotransfected at a 5:1:1:1 ratio for 4–6 h. The transfected cells were harvested and seeded on 96-well plates at a density of 4 × 10^4^ cells for 24 h. On the day of the assay, the culture media was removed and replaced by 90 μl HBSS buffer (pH 7.4) with 20 mM HEPES and 30 μM coelenterazine (GoldBio). The plate was incubated for 5 min at 37°C, and the bioluminescence resonance energy transfer (BRET) signal was measured using SPARK (Tecan) equipped with BRET1 filters. For all tested GPCRs, the BRET signal was measured 10 times before adding ligands or illumination. Blue light illumination (470 nm) was applied to OptoXRs using an LED light source. For ligand-activated GPCRs, 10 μl of the appropriate agonist (e.g., dopamine for DRD2, salvinorin B for KORD) was added to each well to achieve a total volume of 100 μl, immediately before the BRET measurements. The BRET ratios were calculated as the emission at 530 nm divided by the emission at 485 nm. Net BRET values were obtained by comparing the BRET ratios of cells expressing both the donor (Rluc8) and acceptor (mCitrine) to the ones of control cells expressing the donor only:

$$\mathrm{Transduction}\;\mathrm{coefficient}=\mathrm{Log}(E_{\max}/EC_{50}).$$

Transduction coefficient values were determined by normalizing the net BRET values to the maximal response observed for each Gαi/o subtype and calculating the log of the ratio of *E*_max_ to EC_50_ (Kenakin et al., 2012).

### Cell viability assay

The EZ-Cytox assay kit (Daeillab) was used to measure the cytotoxicity of blue light illumination. Cells were seeded on 96-well plates at a density of 2 × 10^4^ cells/ml in a volume of 100 μl/well. After 1 h of illumination, 10 μl of water-soluble tetrazolium salt reagent solution was added to each well, and the plates were incubated for 1 h at 37°C. The absorbance of the living cells was revealed at 450 nm using a Synergy H1 microplate reader (BioTek Instruments).

### Animals

Wild-type (WT) C57BL/6J mice (male, 6–8 weeks) and DRD2-Cre mice with WT littermates [Stock Tg(Drd2-Cre) ER44Gsat/Mmucd, MMRRC; female, 6–8 weeks] were used in experiments. Animals were kept in standard laboratory cages in a temperature (22°C) and humidity (30–60%) regulated facility with a 12 h light/dark cycle. Food and water were provided *ad libitum* according to KIST guidelines. The animal study was approved by the institutional animal care and use committee of KIST (Approval Number 2020-050).

### Stereotaxic injection and cannula implantation

The following viral vectors were used in the optogenetic experiment: AAV8-CamKIIa-Cre [titer, 4.4 × 10^12^ genome copies (GC)/ml, University of North Carolina], and AAVDJ-EF1a-DIO-OptoDRD2-EYFP (titer, 8.8 × 10^12^ GC/ml, KIST Virus Facility). We injected AAV5-EF1a-DIO-EYFP (titer, 6.5 × 10^12^ GC/ml, University of North Carolina) with AAV8-CamKIIa-Cre virus for control groups in Figure 6 and Extended Data Figure 7-1. Surgeries for viral injection and optical fiber implantation were performed using stereotactic equipment (Neurostar), and anesthesia was performed on animals using an isoflurane/oxygen mixture (1–3% at 1 L/min). Moreover, 2 μl of a 1:1 mixture of OptoDRD2 and CamKIIa-Cre viruses (2 μl of a 1:1 mixture of EYFP and CamKIIa-Cre viruses for control groups) was injected into the right lateral globus pallidus (LGP) of each mouse, and a fiber-optic probe (Doric Lenses) was implanted into the same region after 2 weeks of recovery. Stereotactic coordinates used were AP, −0.22; ML, +1.75; and DV −3.75 as measured from the skull surface.

For optogenetic modulation of caudate–putamen (CPu) in Figure 5, we prepared AAV-EF1a-DIO-OptoDRD2-EYFP viruses (titer, 8.8 × 10^12^ GC/ml, KIST Virus Facility) and injected them (1 μl) in the right CPu of either DRD2-Cre (TG) or wild-type (WT) littermates, and a fiber-optic probe (Doric Lenses) was implanted into CPu region after 2 weeks of recovery. Stereotactic coordinates used were AP: +1.0 ML: +1.5 DV: −2.5 as measured from the skull surface.

### Open-field test and calculation of behavioral metrics

Behavior tests were conducted a week after probe implantation (3 weeks after viral injection), using a 50 cm × 50 cm open-field constructed from white opaque acrylic, placed inside a room with sound attenuating panels. The activity of mice placed inside was recorded using an overhead camera, illuminated by an adjacent fluorescent bulb at 40 lx. Optogenetic stimulation was given by a 473 nm crystal laser (Changchun New Industries). Light intensity was adjusted to 10 and 14 mW (Thorlabs) at the fiber tip for striatal and LGP-injected animals, respectively. Light stimulation was at 20 Hz in 5 ms pulses for five 15 s intervals separated by 45 s for the former, and at 10 Hz, 10 ms stimulation was given at 5–10 and 15–20 min during a 20 min trial for the latter. Behavior data analysis was performed on video data acquired using tracking software (EthoVision XT, Noldus). Velocity (*v*) and acceleration (*a*) were calculated from parameters offered by the EthoVision XT tracking software. Meanwhile, “mobility” was defined as when the animal's binned velocity exceeded 0.01 m/s. Start points for continuous bouts of mobility within allotted stimulation trials were defined as mobility initiation.

### Immunostaining

The brains were removed and fixed in paraformaldehyde overnight. Brain slices (40 μm thickness) were permeabilized in 0.5% Triton X-100 in PBS (PBST) for 20 min. Blocking was performed in 10% normal donkey serum in 0.3% PBST for 1 h, after which the samples were incubated with 1:250 rabbit anti-GFP (Cell Signaling Technology, catalog #2555) and 1:200 mouse anti-CamKIIα (Invitrogen, MA1-048) in 0.3% PBST overnight at 4°C. Once washed in 1× PBS, the samples were then placed with 1:400 secondary antibodies, Alexa Fluor-conjugated 488 and 594 (Abcam, ab150077, ab150116), in 0.3% PBST for 1 h at RT. The slices were mounted with Vectashield mounting medium containing 4′,6-diamidino-20phenylindole (DAPI, H-1500; VectorLabs). Confocal images were acquired with LSM800 confocal microscope (Carl Zeiss).

### Statistical analysis

*P* and degrees of freedom (df) were calculated using a two-tailed Student's *t* test and one, two-way ANOVA with Tukey and Dunnett's multiple comparison (Excel or GraphPad Prism 8) for continuous variables, following confirmation of normality calculated by the Shapiro–Wilk test calculator (GraphPad Prism 8).

## Results

### Design of OptoDRD2

Rhodopsin utilizes 9-*cis*-retinal as a chromophore which is isomerized from *cis* to *trans* upon the illumination of blue light (Wald, 1968). This light-induced isomerization of the retinal triggers the active conformation of rhodopsin, which in turn recruits Gαt protein and initiates the Gαt-related downstream pathways (Nakamichi and Okada, 2006; Gao et al., 2019; Fig. 1*A*, left). In contrast, upon the binding of dopamine, DRD2 changes its conformation to interact with Gαi/o, mediating the Gαi/o signaling pathways (Wong, 2003; Katritch et al., 2013; Yin et al., 2020; Fig. 1*A*, middle). The α5 helix of the recruited Gαt or Gαi/o protein contacts with the intracellular loop (ICL) regions of rhodopsin or DRD2, respectively (Gao et al., 2019; Yin et al., 2020; Fig. 1*A*), thus initiating specific downstream signaling pathways related to each Gα type.

To control the DRD2 activation with spatiotemporal resolutions, OptoDRD2 was constructed by combining extracellular loops and transmembrane domains of rhodopsin and the ICL regions from DRD2, and an EYFP was fused to the C terminal to verify its expression and proper cellular localization. We designed this light-sensitive chimeric DRD2 through sequence alignment between bovine rhodopsin and human DRD2 based on GPCR database (Munk et al., 2016) and UniProt (UniProt, 2021; Fig. 1*B*). Both rhodopsin and DRD2 are GPCRs sharing the structure of seven transmembrane domains (Katritch et al., 2013), and AlphaFold (Jumper et al., 2021) predicted that OptoDRD2 can be translated as a chimeric protein (Fig. 1*A*, right). Indeed, the constructed OptoDRD2 was expressed and correctly localized at the plasma membrane and intracellular organelles in HEK293A cells (Fig. 1*C*), indicating successful protein folding and trafficking of OptoDRD2. This localization pattern of OptoDRD2 was consistent with the known features of DRD2 (Takeuchi and Fukunaga, 2003; Lee et al., 2017). As OptoDRD2 contains the residues for the binding to the retinal from rhodopsin as well as the residues for the interaction with Gαi/o protein (Fig. 1*A*, right), we expected that the illumination on OptoDRD2 would initiate the Gαi/o-mediated DRD2 signaling pathways.

### Characterization of OptoDRD2

**Figure 2.**
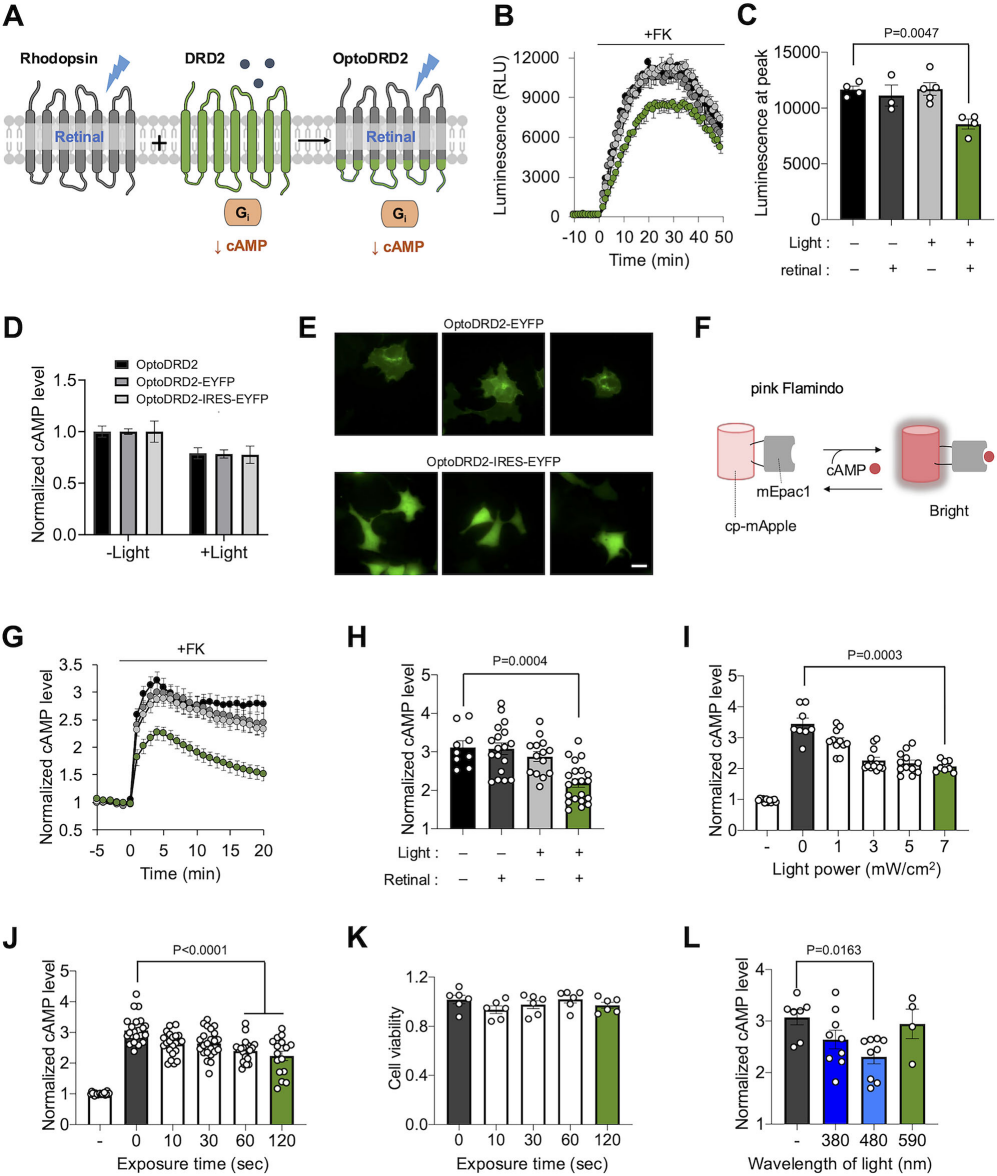
Characterization of OptoDRD2. ***A***, OptoDRD2, generated by the fusion of rhodopsin and DRD2, can induce the DRD2-like signaling pathways in response to blue light, i.e., decreasing the level of cAMP. ***B***, ***C***, Time course of luminescence (***B***) and the maximum luminescence (***C***) of GloSensor in the HEK293A cells expressing OptoDRD2 after the treatment of forskolin (FK, 10 μM) in the following conditions: −light/−retinal (black, *n* = 4); −light/+retinal (dark gray, *n* = 3); +light/−retinal (light gray, *n* = 5); +light/+retinal (green, *n* = 4). A customized LED device was developed for optical stimulation. See Extended Data Figures 2-1 and 2-2 for more details. *n* is the number of wells, and each well contains 2 × 10^4^ cells. Data are shown as means ± SEM. *F* = 9.060, df*n* = 3, df*d* = 13; *p* = 0.9145 (−light/−retinal vs −light/+retinal); *p* = 0.9998 (−light/−retinal vs +light/−retinal); *p* = 0.0047 (−light/−retinal vs +light/+retinal; one-way ANOVA followed by Tukey's multiple-comparisons test). ***D***, Normalized cAMP levels in the cells expressing OptoDRD2, OptoDRD2-EYFP, or OptoDRD2-IRES-EYFP under the treatment of FK (10 μM) without (*n* = 4, 4, 8) or with light (*n* = 4, 6, 7). Data are shown as means ± SEM. *F* = 0.001214, df*n* = 2, df*d* = 3; *p* = 0.9998 (one-way ANOVA followed by Tukey's multiple-comparisons test to control). ***E***, Representative images of OptoDRD2-EYFP (top panel) and OptoDRD2-IRES-EYFP (bottom panel). Scale bar, 20 μm. ***F***, Schematic of a cAMP biosensor, pink-Flamindo (pF). ***G***, ***H***, Red fluorescence of cpmApple in the pink-Flamindo increases upon cAMP binding to Epac1. See Extended Data Figure 2-3 for more details. Time course of fluorescence in ***G*** and the fluorescence at the maximum of the response in ***H*** of pF in the HEK293A cells expressing OptoDRD2 after the treatment of forskolin (FK, 10 μM) in the following conditions: −light/−retinal (black, *n* = 9); −light/+retinal (dark gray, *n* = 18); +light/−retinal (light gray, *n* = 14); +light/+retinal (green, *n* = 21). Data are shown as means ± SEM. *F* = 11.36, df*n* = 3, df*d* = 58; *p* = 0.9986 (−light/−retinal vs −light/+retinal); *p* = 0.7279 (−light/−retinal vs +light/−retinal); *p* = 0.0004 (−light/−retinal vs +light/+retinal; one-way ANOVA followed by Tukey's multiple-comparisons test). ***I***, The pF levels representing cAMP levels upon illumination with different light power in the cells expressing OptoDRD2 under the treatment of FK (10 μM). The pF levels were normalized by the level of negative control without illumination (−; *n* = 12, 8, 11, 12, 12, 8). Data are shown as means ±  SEM. *F* = 5.426, df*n* = 4, df*d* = 50; *p* = 0.4925 (0 vs 1 mW); *p* = 0.2914 (0 vs 3 mW); *p* = 0.1813 (0 vs 5 mW); *p* = 0.0003 (0 vs 7 mW; one-way ANOVA followed by Tukey's multiple-comparisons test). ***J***, Normalized cAMP levels in the cells expressing OptoDRD2 after the illumination of light for different exposure times (*n* = 21, 23, 22, 23, 22, 16). Data are shown as means ± SEM. *F* = 10.25, df*n* = 4, df*d* = 101; *p* = 0.0090 (vs 10 s); *p* = 0.0133 (vs 30 s); *p* < 0.0001 (vs 60 s); *p* < 0.0001 (vs 120 s; one-way ANOVA followed by Tukey's multiple-comparisons test). ***K***, EZ-Cytox assay of the cells expressing OptoDRD2 after illumination (7 mW/cm^2^) for different exposure times. Data are shown as means ± SEM (*n* = 6), *n* is the number of wells and each well contains 2 × 10^4^ cells. *F* = 1.329, df*n* = 4, df*d* = 25; *p* = 0.2866 (one-way ANOVA followed by Tukey's multiple-comparisons test to control). ***L***, Normalized cAMP levels in the cells expressing OptoDRD2 after illumination with different wavelengths of light (*n* = 7, 9, 9, 4). Data are shown as means ±  SEM. *F* = 3.998, df*n* = 3, df*d* = 25; *p* = 0.4847 (- vs 380 nm); *p* = 0.0199 (- vs 480 nm); *p* > 0.9999 (- vs 590 nm; one-way ANOVA followed by Tukey's multiple-comparisons test).

We next examined whether OptoDRD2 can induce the Gαi/o-mediated signaling pathway upon illumination of blue light (Civelli et al., 1993). For example, we first utilized GloSensor to confirm the decreased cAMP level via Gαi/o-mediated signaling pathways (Fig. 2*A*). GloSensor is a circularly permuted luciferase that is engineered to increase its activity upon the cAMP binding, thus reporting the cAMP levels by the change in luminescence (Fan et al., 2008). We treated forskolin (FK, 10 μM) to activate adenylyl cyclase and increase the cAMP level and applied blue light (7 mW/cm^2^ for 60 s) to check whether OptoDRD2 can decrease the cAMP level. The results showed that illumination on OptoDRD2 can significantly decrease the cAMP level (Fig. 2*B,C*). We also compared the cAMP level after illumination on OptoDRD2 with or without retinal. Our results confirmed that illumination on OptoDRD2 without retinal is not functional (Fig. 2*B,C*), suggesting that the light-induced isomerization of the retinal is required for the function of OptoDRD2 decreasing the cAMP level. Therefore, the chimeric receptor OptoDRD2 can function like a blue light-responsive DRD2. To ensure that the C-terminal EYFP did not interfere with OptoDRD2 functionality, we generated two additional constructs, OptoDRD2 without EYFP and OptoDRD2-IRES-EYFP. The cells expressing OptoDRD2, OptoDRD2-EYFP, or OptoDRD2-IRES-EYFP exhibited comparable basal cAMP levels and similar reductions in cAMP upon illumination. These findings demonstrate that the fluorescent protein tagging does not alter OptoDRD2 function (Fig. 2*D,E*), consistent with previous observations in other OptoXRs and similar optogenetic tools (Airan et al., 2009; Gunaydin et al., 2014; Siuda et al., 2015).

10.1523/JNEUROSCI.1473-24.2024.f2-1 Figure 2-1 **Development of the customized LED device. *A,*** Design of the LED device for illuminating the cells expressing OptDRD2 from the top side of a culture dish. ***B****,* The LED array and wireless controllers are located in the lower panel of the device and the upper panel contains potentiometers, power supply connector and holder, and program channels. ***C,*** Workflows of the power supply, wireless switch, LED array, and wireless controller. Download Figure 2-1, TIF file.

10.1523/JNEUROSCI.1473-24.2024.f2-2 Figure 2-2 Optic power of the LED device according to rotation angle of the potentiometer. Download Figure 2-2, TIF file.

**Figure 3.**
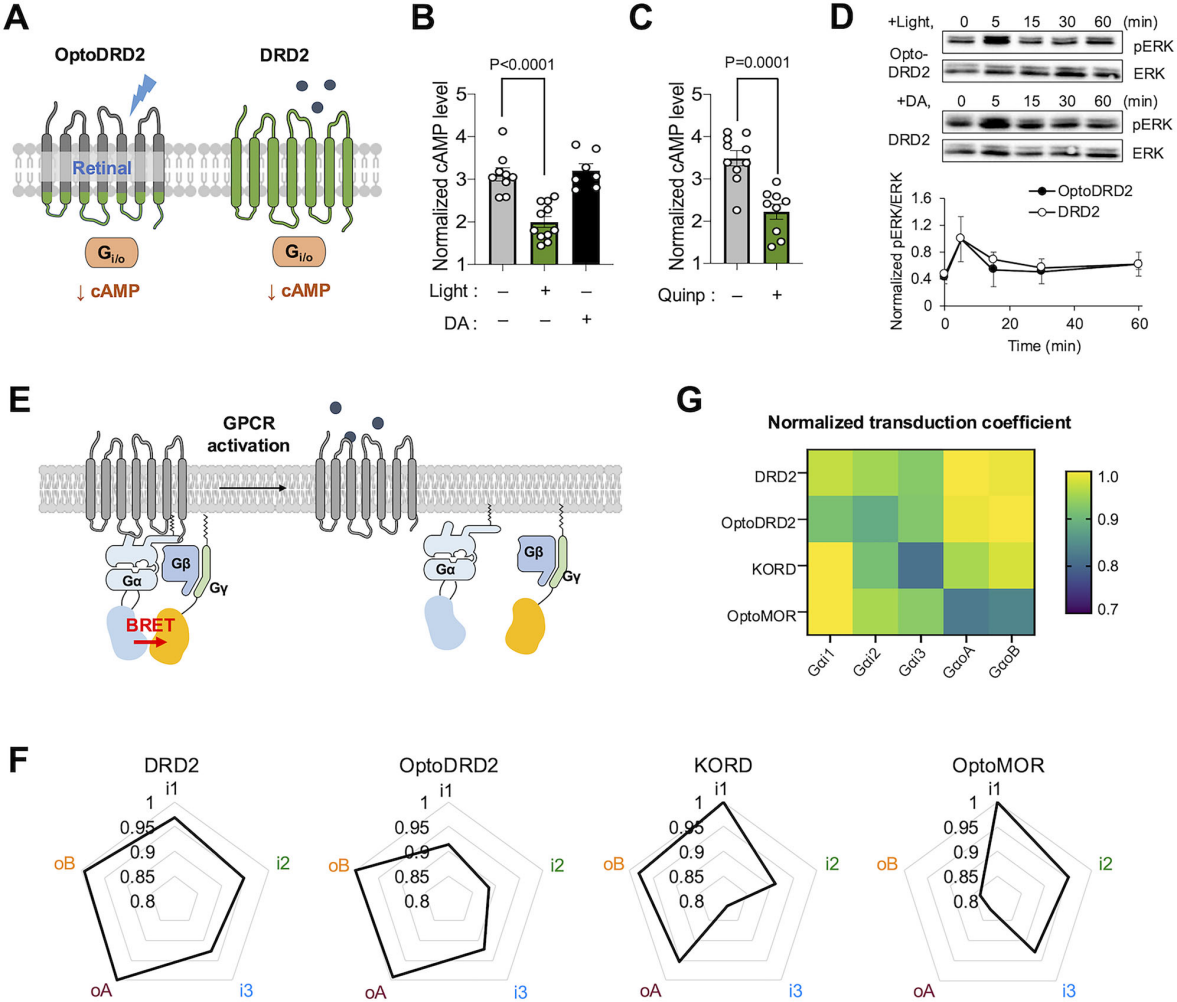
OptoDRD2 can induce the DRD2-like signaling pathways upon illumination. ***A***, Schematic of OptoDRD2 and DRD2. ***B***, Normalized cAMP levels in the cells expressing OptoDRD2 under the treatment of FK (10 μM) without or with light (*n* = 11, 9) or with the treatment of dopamine (10 μM, *n* = 7). Data are shown as means ± SEM. *p* < 0.0001, *t* = 5.606, df = 18 (−light vs +light); *p* = 0.7040, *t* = 0.3879, df = 14 (−light vs +DA; two-tailed, unpaired *t* test). ***C***, Normalized cAMP levels in the cells expressing OptoDRD2 under the treatment of FK (10 μM) without or with the treatment of quinpirole (*n* = 10, 9). Data are shown as means ± SEM. *p* = 0.0001, *t* = 4.963, df = 17 (two-tailed, unpaired *t* test). ***D***, Time courses of ERK phosphorylation in the cells expressing OptoDRD2 after light illumination or in the cells expressing DRD2 after the dopamine treatment to DRD2 (10 μM; *n* = 3). Time courses of ERK phosphorylation by other Gαi/o-activating tools are shown in Extended Data Figure 3-1. The graph shows the normalized ratio of phosphorylated ERK to total ERK levels. Normalized values were calculated using the maximum value of each group. Data are shown as mean ± SEM. ***E***, Schematic of BRET-based G-protein coupling assay, TRUPATH. ***F***, Radar plots representing the normalized transduction coefficient values of DRD2, OptoDRD2, KORD, and OptoMOR across different Gαi/o subtypes (i1, i2, i3, oA, oB). Each axis represents a different Gαi/o subtype and the distance from the center indicates the coupling strength. Dose–response curves are shown in Extended Data Figure 3-2. See Extended Data Figure 3-2 for more details. ***G***, Heatmap illustrates the normalized transduction coefficient values for DRD2, OptoDRD2, KORD, and OptoMOR, across different Gαi/o subtypes (i1, i2, i3, oA, oB). The color gradient represents the level of selectivity, with yellowish colors indicating higher coupling selectivity. The transduction coefficient values were determined by normalizing the net BRET values to the maximal response observed for each Gαi/o subtype and calculating the log of the ratio of *E*_max_ to EC_50_ (Kenakin et al., 2012).

10.1523/JNEUROSCI.1473-24.2024.f2-3 Figure 2-3 **Visualization of the cAMP levels after the activation of OptoDRD1 and OptoDRD2.** Representative images of the pink Flamindo (pF) in the cells expressing OptoDRD2 without or with illumination, under the treatment of 10 μM forskolin. Scale bar, 20 μm. Download Figure 2-3, TIF file.

For the characterization of OptoDRD2, we also utilized pink-Flamindo (pF), a circular permuted red fluorescent protein-based cAMP sensor which increases its fluorescence upon the cAMP binding (Harada et al., 2017; Fig. 2*F*). After the treatment of FK (10 μM) and illumination of blue light (7 mW/cm^2^ for 60 s) on OptoDRD2, we detected a decrease in red fluorescence (Fig. 2*G,H*) indicating the OptoDRD2-mediated reduction in the cAMP levels. Again, retinal was required for the function of OptoDRD2 (Fig. 2*G,H*), confirming the light-induced activation mechanism of OptoDRD2.

We confirmed that the activation of OptoDRD2 is dependent on the power and exposure time of blue light, and OptoDRD2 can be efficiently activated by the illumination of 7 mW/cm^2^ of blue light for 60 s (Fig. 2*I,J*) without affecting cell viability (Fig. 2*K*). We also confirmed that 480 nm of blue light efficiently induces the activation of OptoDRD2 (Fig. 2*L*). These results suggest that we can finely control the activation of OptoDRD2 by blue light.

### Illumination of OptoDRD2 induces the DRD2-like downstream signaling pathways

To verify the functional similarity between OptoDRD2 and DRD2, we first compared the capacity decreasing the cAMP level of OptoDRD2 and DRD2 (Fig. 3*A*). Illumination of blue light on OptoDRD2 (7 mW/cm^2^ for 60 s) resulted in a reduction of cAMP level (Fig. 3*B*), comparable to that observed when DRD2 was activated by its specific agonist quinpirole (10 μM; Fig. 3*C*). In contrast, the treatment of dopamine (10 μM) did not activate OptoDRD2 inducing no change in the cAMP level (Fig. 3*B*). Next, we examined whether OptoDRD2 can induce the ERK phosphorylation, which demonstrated that OptoDRD2 induces ERK phosphorylation in a manner similar to DRD2 (Fig. 3*D*). While it is a frequently used marker for DRD2 activation (Choi et al., 1999), the ERK phosphorylation can be also induced by other Gαi/o-activating tools (Extended Data Fig. 3-1). Thus, further investigation of DRD2-like events is necessary to validate the functionality of OptoDRD2.

10.1523/JNEUROSCI.1473-24.2024.f3-1 Figure 3-1 **Time course of ERK phosphorylation of Gαi-type GPCRs upon activation.** Time courses of ERK phosphorylation in the cells expressing DRD2 (n = 3) (***A***), OptoDRD2 (n = 5) (***B***), KORD (n = 4) (***C***) and OptoMOR (n = 3) (***D***) after light illumination or ligand treatment (10 μM dopamine for DRD2, 10 μM SalB for KORD). The graph shows the normalized ratio of phosphorylated ERK to total ERK levels. Data are shown as mean ± s.e.m. *F* = 8.123, *df*n = 4, *df*d = 10; p = 0.0012 (0  min vs. 5  min); p = 0.1485 (0  min vs. 15  min); p = 0.7331 (0  min vs. 30  min); p = 0.4279 (0  min vs. 60  min); (*A*, DRD2); *F* = 2.498, *df*n = 4, *df*d = 20; p = 0.0423 (0  min vs. 5  min); p = 0.9870 (0  min vs. 15  min); p = 0.9995 (0  min vs. 30  min); p = 0.9299 (0  min vs. 60  min); (*B*, OptoDRD2); *F* = 5.419, *df*n = 4, *df*d = 15; p = 0.0434 (0  min vs. 5  min); p = 0.7735 (0  min vs. 15  min); p = 0.7960 (0  min vs. 30  min); p = 0.5937 (0  min vs. 60  min); (*C*, KORD); *F* = 2.767, *df*n = 4, *df*d = 10; p = 0.0382 (0  min vs. 5  min); p = 0.8553 (0  min vs. 15  min); p = 0.6348 (0  min vs. 30  min); p = 0.9671 (0  min vs. 60  min); (*D*, OptoMOR). (one-way ANOVA followed by Dunnett’s multiple comparison test). Download Figure 3-1, TIF file.

10.1523/JNEUROSCI.1473-24.2024.f3-2 Figure 3-2 **Different Gα subtype coupling profiles of Gαi/o type GPCRs.** Dose-response curves of Gαi/o subtypes (i1, i2, i3, oA, oB) for DRD2 (***A***), OptoDRD2 (***B***), KORD (***C***), and OptoMOR (***D***) in response to light stimulation or drug treatment (dopamine for DRD2, SalB for KORD). Data are shown as mean ± s.e.m. (n = 4), n is the number of wells and each well contains 4 × 10^4^ cells. Download Figure 3-2, TIF file.

It has been known that activated GPCRs recruit a particular type of Gα proteins, e.g., Gαs, i/o, q/11, 12/13, and the recruited Gαβγ proteins then dissociated into Gα and βγ, mediating G-protein–specific downstream signaling pathways (Wettschureck and Offermanns, 2005; Harding et al., 2018). Gα proteins comprise several subtypes, each mediating distinct signaling pathways and unique cellular responses (Inoue et al., 2019; Avet et al., 2022; Hauser et al., 2022). To investigate the Gα subtype specificity for OptoDRD2, we employed the TRUPATH system which measures the dissociation between Gα and Gβγ proteins by bioluminescence resonance energy transfer (BRET; Fig. 3*E*).

In the TRUPATH assay for OptoDRD2, we measured the BRET changes in response to different doses of stimulation (Fig. 3*F* and Extended Data Fig. 3-2), and then the Gα subunit specificity was represented by transduction coefficient calculated from *E*_max_ and EC_50_ (Kenakin et al., 2012). We observed similar Gα subunit specificity between OptoDRD2 and DRD2 (Gαi1, i2, i3 < GαoA, oB) while another Gαi-type optogenetic tool, OptoMOR, specifically activates Gαi1 (Fig. 3*G*). We also observed distinct Gα subtype specificity for the Gαi-type chemogenetic tool, KORD (Fig. 3*G*). These results suggest that each Gαi-type GPCR activates unique Gαi-subtype profiles, which may result in different combinations of downstream signaling pathways (Jiang and Bajpayee, 2009; Villaseca et al., 2022). Therefore, for the induction of a more DRD2-like response, it is required to contain the intracellular parts of DRD2 in the design of OptoDRD2.

**Figure 4.**
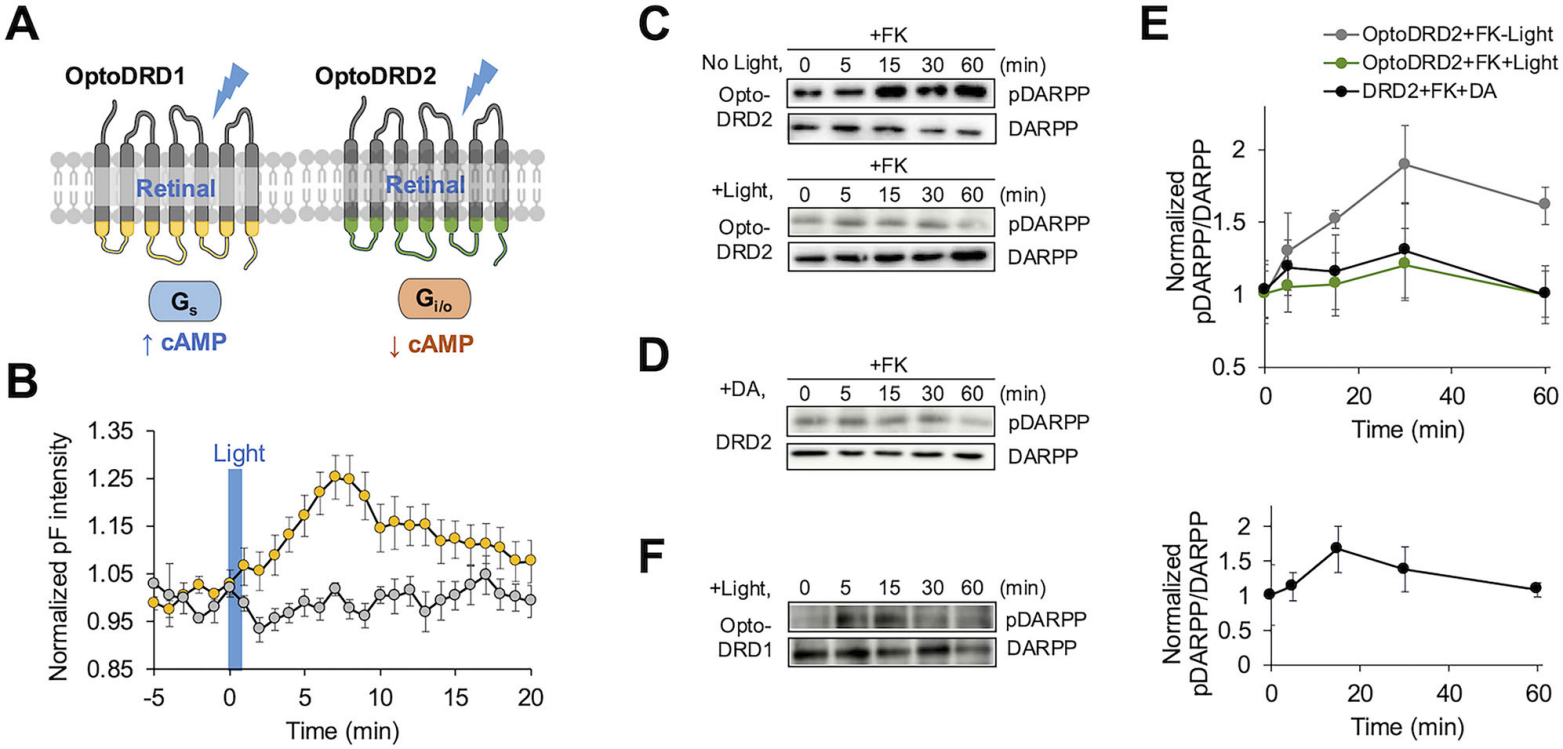
OptoDRD2 triggers distinct downstream signaling from OptoDRD1. ***A***, Schematic of OptoDRD1 and OptoDRD2. ***B***, Time course of cAMP levels in the cells expressing OptoDRD1 in response to illumination without (gray, *n* = 4) or with retinal (yellow, *n* = 10). ***C***, ***D***, Time course of the DARPP-32 phosphorylation in the cells expressing OptoDRD2 under the treatment of FK (1 μM) without or with light stimulation for 60 s (*n* = 4 and 3; ***C***) and in the cells expressing DRD2 which also treatment of FK (1 μM) with dopamine (10 μM; *n* = 5; ***D***). ***E***, The graph shows the normalized ratio of phosphorylated DARPP-32 to total DARPP-32 levels. Normalized values were calculated using the minimum value of each group. Data are shown as mean± SEM. ***F***, Time course of the DARPP-32 phosphorylation in the cells expressing OptoDRD1 upon blue light illumination for 60 s (*n* = 3). The graph shows the normalized ratio of phosphorylated DARPP-32 to total DARPP-32 levels. Normalized values were calculated using the minimum value of each group. Data are shown as mean ± SEM.

We next tested whether OptoDRD2 induces the DRD2-like signaling distinct from OptoDRD1 (Gunaydin et al., 2014; Fig. 4*A*). OptoDRD1 is composed of the light-sensing domains of rhodopsin and the ICL regions of DRD1; thus, it can recruit Gαs protein upon activation inducing the increase of cAMP level. Indeed, we can observe the increased intensity of pink-Flamindo upon light stimulation in the cells expressing OptoDRD1 supplemented with retinal (Fig. 4*B* and Extended Data Fig. 4-1). These results demonstrated that OptoDRD1 activates Gαs protein to elevate cAMP levels, in contrast to OptoDRD2 (Fig. 2). This confirms that OptoDRD1 and OptoDRD2 can selectively activate their corresponding G-protein–like signaling pathways.

We further examined the downstream signaling molecule of the dopaminergic pathway, DARPP-32, in response to OptoDRD1 and OptoDRD2. It has been known that the activation of DRD1 increases the level of cAMP and activates PKA, which then phosphorylates DARPP-32 on Thr34 (Nishi et al., 2000). This event can be downregulated by the activation of DRD2 (Stoof and Kebabian, 1981; Bateup et al., 2008). Indeed, the cells expressing OptoDRD2 inhibited the FK-induced increase of pDARPP-32 level upon illumination (Fig. 4*C,E*), which is consistent with the effects observed following DRD2 activation (Fig. 4*D,E*). In contrast, OptoDRD1 induced the phosphorylation of DARPP-32 (Fig. 4*F*). Therefore, OptoDRD2 is capable of causing DRD2-like downstream events distinct from OptoDRD1.

### Optogenetic stimulation of OptoDRD2 in the striatum of mice brain causes the changes in motor behavior

**Figure 5.**
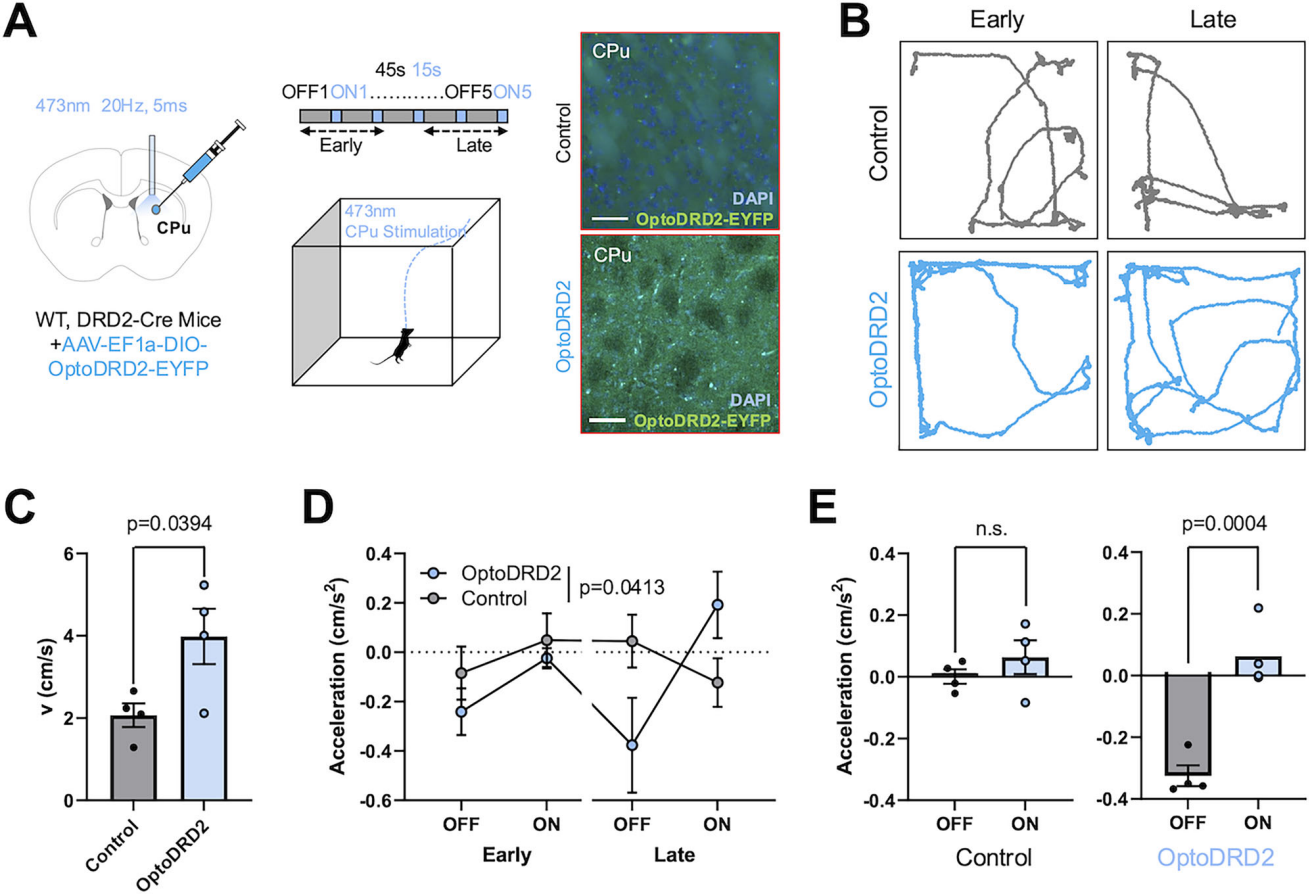
Optogenetic stimulation of OptoDRD2 in the striatum of mice brain causes changes in motor behavior. ***A***, Left, Schematic diagram (left) of virus injection for open-field test (middle) in the caudate–putamen (CPu) region of control (WT; *n* = 4) and OptoDRD2-expressing mice (DRD2-Cre; *n* = 4). Right, Representative expression of OptoDRD2-EYFP (green) and DAPI (blue) in right CPu. Scale bar, 50 μm. ***B***, Representative movement track in the open field of control and OptoDRD2 groups during early (on, off 1–2) and late (on, off 4–5) trials. ***C***, Overall velocity during “off 5” to “on 5” trials of control and OptoDRD2 mice. Data are shown as means ± SEM. *p* = 0.0394, *t* = 2.624, df = 6 (two-tailed, unpaired *t* test). ***D***, Diverging average acceleration in the open field from early (off, on 1–2) to late (off, on 4–5) trials for control and OptoDRD2 mice. Data are shown as means ± SEM. *F *= 3.370, df*n *= 3, df*d* = 24; *p* = 0.0350 (two-way ANOVA test). ***E***, Acceleration values throughout the trial for all off and on periods of control and OptoDRD2 animals. Data are shown as means ± SEM. *p*_control _= 0.4120, *t*_control _= 0.9504, df_control _= 3; *p*_OptoDRD2 _= 0.0004, *t*_OptoDRD2 _= 18.40, df_OptoDRD2 _= 3 (two-tailed paired *t* test).

We next assessed whether OptoDRD2 can be used as an optogenetic tool to activate the DRD2-like pathways in the brains of living animals. The existence of DRD2 in the striatum (caudate–putamen, CPu) and its motor function through the indirect pathway of basal ganglia have been well-documented (Tecuapetla et al., 2014; J. Kim et al., 2017). Thus, we prepared AAV-EF1a-DIO-OptoDRD2-EYFP viruses and injected them in the right CPu of either DRD2-*Cre* (TG) or wild-type (WT) littermates. To investigate the effect of OptoDRD2 on motor functions after optogenetic stimulation, we implanted optic fibers into the dorsal CPu 2 weeks after the virus infection. After another week, an open-field test was conducted on the light stimulation (Fig. 5*A*), in accordance with previous studies that applied optogenetic stimulation on the striatum region (Gunaydin et al., 2014; Grimm et al., 2021).

Interestingly, light stimulation induced gradual increases in movement at OptoDRD2-expressing mice from early to late trials (Fig. 5*B*). The velocity was also significantly increased during the late trial periods (Fig. 5*C*). We next investigated differences in other movement parameters, particularly vigor or acceleration which are known to be controlled by dopaminergic circuits in basal ganglia (da Silva et al., 2018). It was clearly evident that acceleration increased during the “on” phase from the level of its “off” phase in OptoDRD2-expressing mice, but not in control mice (Fig. 5*D,E*). Regarding downstream signaling of DRD2, the activation of OptoDRD2 would “decrease” the excitability of striatal neurons in the indirect circuits (Ericsson et al., 2013), therefore resulting in an increased tendency toward mobility. These results confirmed that OptoDRD2 can specifically mediate the known functions of DRD2 (Kravitz et al., 2010), implying that OptoDRD2 can be further applied to investigate novel DRD2 functions at particular cell types or regions in the dopaminergic circuits of the brain.

### OptoDRD2 as a tool to investigate novel function of DRD2 in specific brain regions

**Figure 6.**
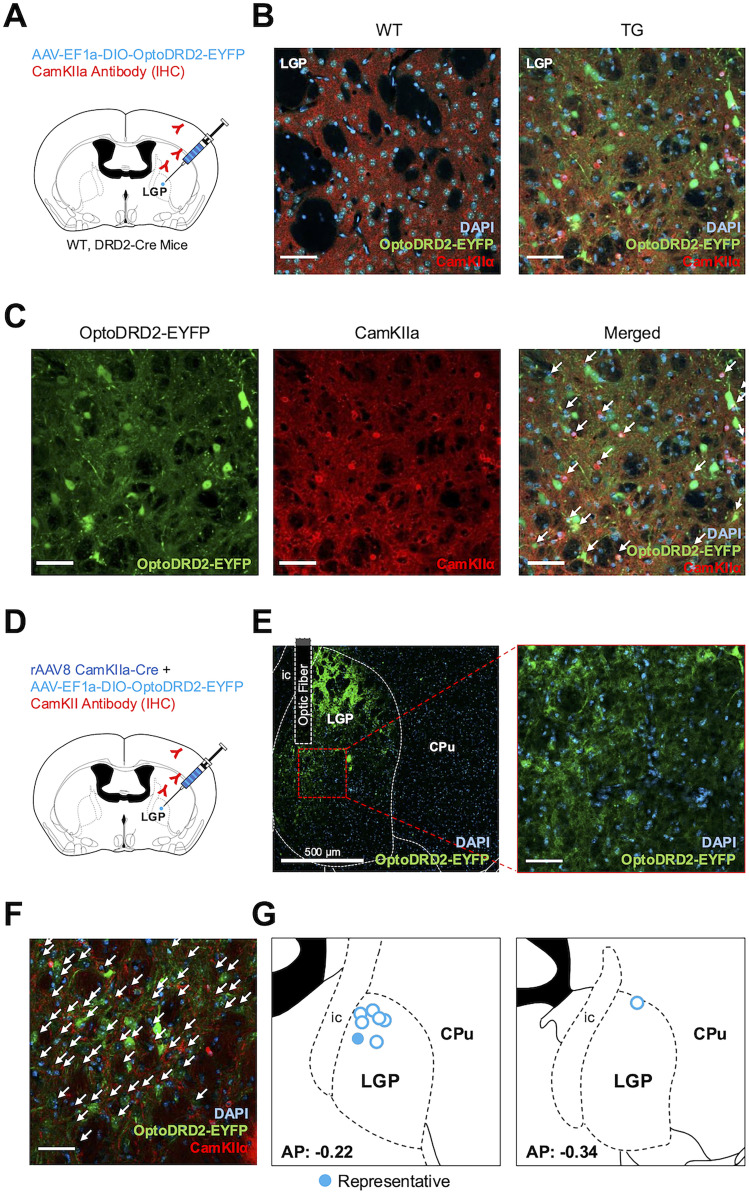
CamKII(+) neurons in LGP regions can express OptoDRD2 and are targeted with optogenetics. ***A***, Schematic diagram of virus injection and immunohistochemistry in LGP. ***B***, Immunohistochemistry in LGP with CamKIIa antibody (red) in WT and DRD2-Cre TG mice. ***C***, Representative confocal images of OptoDRD2-EYFP expression (green) with CamKlla antibody (red) and DAPI (blue) in DRD2-Cre TG mice. The white arrows indicate the colocalization of OptoDRD2 and CamKIIa. ***D***, Schematic diagram of virus injection and immunohistochemistry in LGP. ***E***, OptoDRD2-EYFP expression with fiber implant in right LGP (green) and DAPI expression (blue). ***F***, Immunohistochemistry in LGP with CamKIIa antibody (red). The white arrows indicate the colocalization of OptoDRD2 and CamKIIa. ***G***, Optogenetic fiber tip locations for OptoDRD2 mice are shown in Figures 5 and 6 (*n* = 8). The solid circle indicates a representative image in ***E***. Scale bar, 50 μm (not separately indicated).

We next applied OptoDRD2 to explore DRD2 functions in other brain regions. In particular, we investigated the roles of DRD2 in glutamatergic cell populations of basal ganglia circuitry, based on the recent findings that DRD2 mRNA expression is highly merged with CamKIIa, a marker for excitatory neurons (Saunders et al., 2018), and that its particular cluster population is found within the lateral globus pallidus (LGP; Lein et al., 2007; Uhlén et al., 2015). To confirm the expression of CamKIIa in DRD2-positive LGP neurons, we transfected AAV vectors harboring DIO-OptoDRD2-EYFP in DRD2-*Cre* (TG) or WT mice and performed immunohistochemistry using antibodies against CamKIIa (Fig. 6*A*). We observed the expression of OptoDRD2-EYFP in LGP region of DRD2-*Cre* (TG) mice, but not WT mice (Fig. 6*B*). Furthermore, CamKIIa was highly expressed in the DRD2-positive LGP neurons and colocalized with OptoDRD2-EYFP (Fig. 6*C*). These results suggest that CamKIIa-positive neuron is a novel cell type expressing DRD2 in the LGP region.

To investigate the causal role of DRD2 in CamKIIa-positive neurons, we implemented AAV-EF1a-DIO-OptoDRD2-EYFP with AAV-CamKIIa-Cre in the right LGP region (Fig. 6*D*). We also confirmed the expression of OptoDRD2-EYFP in CamKIIa-positive LGP neurons using immunohistochemistry with a CamKIIa antibody (Fig. 6*E,F*). The functions of these excitatory LGP neurons have not been previously examined, although intrapallidal DRD2 neurons, in general, may attenuate upstream striatal inhibition of LGP (Querejeta et al., 2001). We therefore applied optogenetic stimulation to the mouse brain expressing OptoDRD2 to investigate the unknown functions of DRD2 in the excitatory LGP neurons. Similar to the previous experiment, after the infection of AAV vectors expressing OptoDRD2-EYFP or EYFP, we implanted optogenetic probes in the LGP region and applied optogenetic stimulation to the OptoDRD2-EYFP- or EYFP-expressing mice during open-field tests (Fig. 6*G*).

**Figure 7.**
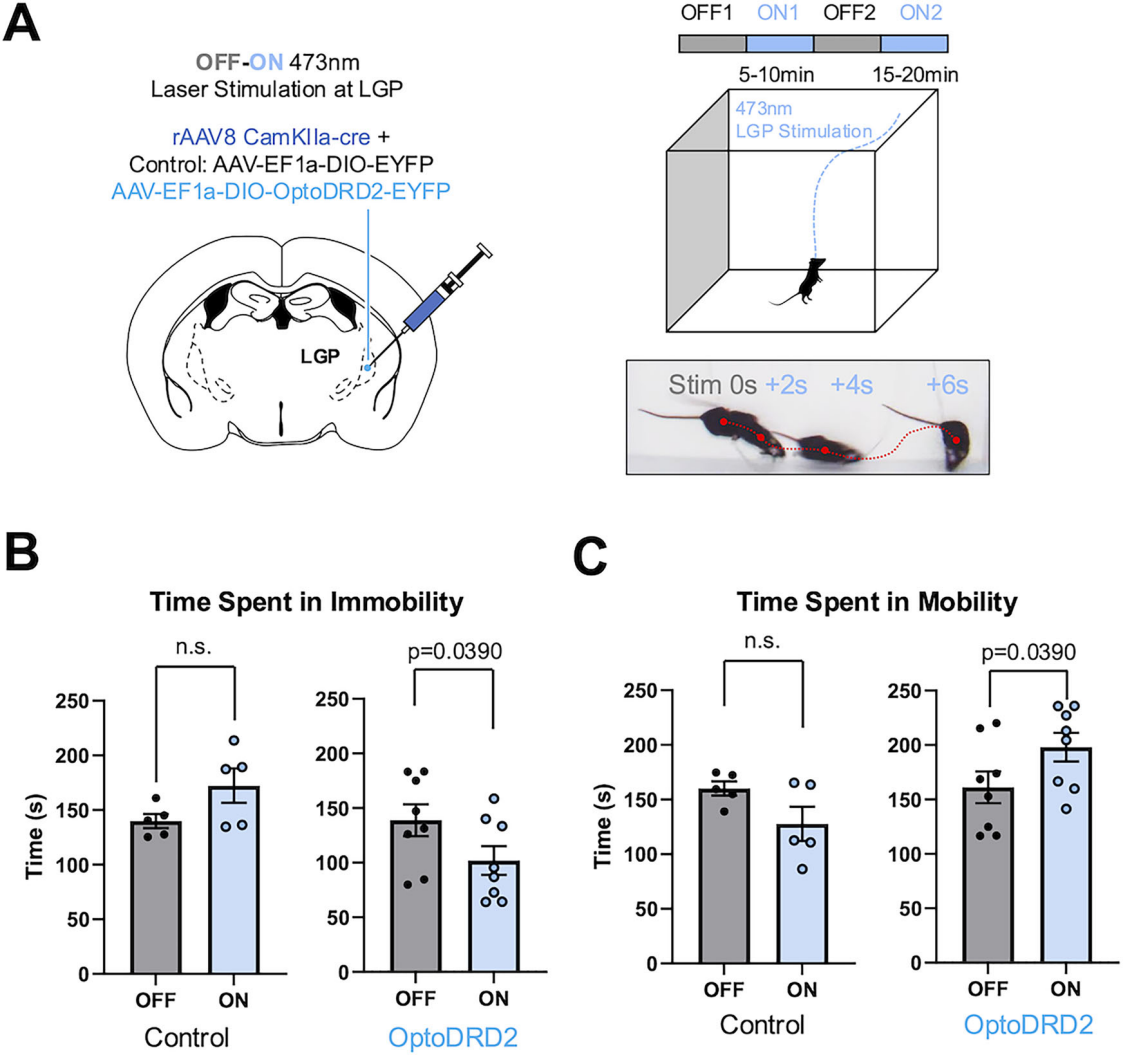
Optogenetic stimulation of CamKII(+) DRD2 neurons in the LGP region expressing OptoDRD2 is correlated with increases in high-velocity movement. ***A***, Schematic of behavior experiment and optogenetic stimulation using control (EYFP-expressing; *n* = 5) and OptoDRD2-expressing mice (*n* = 8). Example of behavior during stimulation initiation. ***B***, Time spent in immobility during “on” and “off” trials, control (left), and OptoDRD2 (right). Data are shown as means ± SEM. *p*_control _= 0.0649, *t*_control _= 2.526, df_control _= 4; *p*_OptoDRD2 _= 0.0390, *t*_OptoDRD2 _= 2.535, df_OptoDRD2 _= 7 (two-tailed paired *t* test). ***C***, Time spent in mobility during “on” and “off” trials, control (left), OptoDRD2 (right). Data are shown as means ± SEM. *p*_control _= 0.0649, *t*_control _= 2.526, df_control _= 4; *p*_OptoDRD2 _= 0.0390, *t*_OptoDRD2 _= 2.535, df_OptoDRD2 _= 7 (two-tailed paired *t* test). See Extended Data Figure 7-1 for more details.

We observed differences in the movement of mice groups between light “off” and “on” sessions (Fig. 7*A*). We further defined mobility as any movement of the animal's center point exceeding 0.01 m/s and analyzed the mobility of OptoDRD2-expressing mice. An increase in the frequency of mobile bouts was observed in mice expressing OptoDRD2 during the light “on” periods (Extended Data Fig. 7-1*A*). This increase also correlated with the increase in total mobility time (Fig. 7*B,C*), while no significant difference was observed in average velocity (Extended Data Fig. 7-1*B*). We also found acceleration was amplified during “on” trials in the OptoDRD2 group (Extended Data Fig. 7-1*C*). This effect on acceleration highlights the potential importance of DRD2 in LGP excitatory neurons for facilitating movement vigor, which may in turn lead to more mobile states. Therefore, OptoDRD2 can be applied to selectively activate DRD2-like signaling and explore the uncovered potential functions of DRD2 in specific cell types or brain regions.

10.1523/JNEUROSCI.1473-24.2024.f7-1 Figure 7-1 **Optogenetic stimulation of CamKII(+) DRD2 neurons in LGP region expressing OptoDRD2 induces increased movement vigor. *A,*** Frequency of mobility during ‘on’ and ‘off’ session, for control (n = 5) (left), OptoDRD2 (n = 8) (right). Data are shown as means ± s.e.m. p_control_ = 0.1351, t_control_ = 1.868, *df*_control_ = 4; p_OptoDRD2_ = 0.0300, t_OptoDRD2_ = 2.714, *df*_OptoDRD2_ = 7. (two-tailed paired t-test). ***B,*** Average velocity throughout the trial for control (left), OptoDRD2 (right). Data are shown as means ± s.e.m. p_control_ = 0.0677, t_control_ = 2.486, *df*_control_ = 4; p_OptoDRD2_ = 0.1528, t_OptoDRD2_ = 1.604, *df*_OptoDRD2_ = 7. (two-tailed paired t-test). ***C,*** Overall acceleration during on and off trials of OptoDRD2 animals (left), and by individual period (right) (n = 8). Data are shown as means ± s.e.m. p = 0.0202, t = 2.991, *df* = 7 (Overall acceleration); p = 0.0788, t = 2.057, *df* = 7 (Trial 1); p = 0.0169, t = 3.117, *df* = 7 (Trial 2). (two-tailed paired t-test). Download Figure 7-1, TIF file.

## Discussion

Optogenetics has profoundly advanced neuroscience by enabling spatiotemporal control of neural activity (Deisseroth et al., 2006). The most widely used optogenetic technique is channelrhodopsin (ChR), a light-gated ion channel which allows the influx of cation ions upon illumination (Nagel et al., 2002; Boyden et al., 2005). ChR is a fast and powerful method to generally activate neurons with light; however, it is limited to induce particular neurotransmission in the complex brain circuits. Because brain functions are finely controlled by combinations of particular neurotransmissions at specific brain regions (Avery and Krichmar, 2017; Granger et al., 2017), optogenetic tools that can selectively control neurotransmissions are necessary to investigate complex brain functions with high spatiotemporal resolution.

During neurotransmission, the released neurotransmitters bind to neurotransmitter receptors at the postsynaptic neurons initiating particular intracellular responses. Because different subtypes of the receptors can induce differential downstream signaling pathways, for example, DRD1 and DRD2 subtypes, the activation of different subtypes of neurotransmitter receptors may result in different cellular responses and animal behaviors (K. M. Kim et al., 2012; B. Kim et al., 2018; Cole et al., 2018). Thus, it has been widely believed that DRD1 and DRD2 facilitate opposite motor outputs, increasing and decreasing motor output, respectively, especially when viewed within simplistic on/off paths of basal ganglia. However, motor functions may be regulated by spatiotemporal activities of combinations of DRD subtypes in the dopaminergic circuits, and it is more complex than this simple notion which indeed has been progressively challenged (Calabresi et al., 2014; J. Kim et al., 2017). Thus, it is important to develop new optogenetic strategies that can precisely control the individual activity of DRD1 and DRD2 at specific brain regions in the dopaminergic circuits to explore their exact functions for motor functions.

Here, we developed OptoDRD2, a novel optogenetic actuator which can selectively activate DRD2-like signaling upon illumination of blue light. OptoDRD2 was constructed by combining the light-sensitive domains of rhodopsin and intracellular functional parts of DRD2. We confirmed that the blue light can finely control the activation of OptoDRD2 and initiate the DRD2-like downstream signaling pathways such as the cAMP decrease and the phosphorylation of ERK. Using the TRUPATH assay, we further verified that OptoDRD2 exhibits Gαi/o subtype specificity most similar to DRD2 among other Gαi-activating tools such as OptoMOR and KORD. This implies the importance of retaining the intracellular components of DRD2 for inducing DRD2-like signaling, as each Gαi/o subtype can activate distinct combinations of downstream signaling pathways (Jiang and Bajpayee, 2009; Villaseca et al., 2022). While OptoDRD2 does not fully replicate the G-protein subtype specificity of DRD2, DRD2 and OptoDRD2 share a common preference for Gαo proteins, enabling DRD2-like signaling pathways by OptoDRD2. Additionally, high expression of Gαo proteins in the brain (Worley et al., 1986) may contribute to the DRD2-like responses induced by OptoDRD2 in vivo. These findings demonstrate the functional similarity between OptoDRD2 and DRD2, while further refinement is needed to improve the Gα subtype specificity of OptoDRD2.

The PORTL system, which uses covalently taggable chemical photoswitches, enabled precise control of endogenous receptor activation through light-mediated ligand switching of the attached ligands (Hetzler et al., 2023). This approach offers significant advantages by targeting endogenous receptors. However, PORTL requires both the overexpression of membrane-targeted enzymes and additional coupling of photoswitchable ligands to these enzymes. In contrast, our fully genetically encoded OptoDRD2 provides significant advantages for in vivo studies. We believe both approaches have unique strengths and will provide valuable insights for the understanding of dopamine receptor signaling in different experimental contexts.

In this study, we expressed OptoDRD2 in the excitatory neurons of LGP and successfully applied the optogenetic stimulation in living mice to investigate the potential role of DRD2 for motor function. DRD2 has been characterized as predominantly inhibitory in its motor function; however, recent studies have highlighted exceptions to the supposed neat categorization of movement-generating or movement-diminishing neuron types segregated in discrete regions in animal and human models (Tecuapetla et al., 2016; Furness et al., 2021), including the example in the basal ganglia. Thus, our discovery of an excitatory DRD2-positive LGP population is surprising, but it is not entirely unprecedented (Calabresi et al., 2014; Park et al., 2022). Rather, the use of OptoDRD2 has enabled a functional dissection of cells coexpressing CamKIIa and DRD2, previously noted from transcriptomic analyses as being represented by the ppp1r1b genetic marker (Saunders et al., 2018). In line with our findings, these ppp1r1b-positive neurons are known to be distributed in LGP (Uhlén et al., 2015) and may act in regulating dopamine-related synaptic plasticity inside basal ganglia (Yagishita et al., 2014) complementing the DRD2's known role in decreasing downstream signals (Zhang et al., 2021). Our findings could hint at an excitatory motor role for DRD2-positive cell-dependent mechanisms, opening potential areas for further study. Ultimately, this application confirmed the tractability and convenience of OptoDRD2 as a neuroscientific tool.

Despite OptoDRD2 being very useful in behavioral neuroscience, effective stimulation of dopamine receptors requires the use of a high-power laser. Therefore, it is important to interpret the data by distinguishing the behavioral changes induced by OptoDRD2 or by the potential effects of visual stimulation from the light. Additionally, it is crucial to consider the potential effects of overexpression which may impact the cellular environment and downstream signaling pathways. Therefore, it is essential to carefully design the expression methods to induce appropriate levels of OptoDRD2 expression and to compare the experimental results with proper controls to ensure accurate interpretation of data.

Currently, both OptoDRD1 and OptoDRD2 are activated by blue light. To utilize OptoDRD2 together with OptoDRD1 in the complex dopaminergic circuits, further efforts are required to diversify the wavelengths of light to activate OptoXRs, possibly through modifications in the light-responsive rhodopsin part of OptoXRs (Janz and Farrens, 2001; Rajamani et al., 2011; Tichy et al., 2022). The simultaneous optogenetic application of these multicolor OptoDRD1 and OptoDRD2 will further reveal the precise roles of DRD1 and DRD2 during dopaminergic transmission in the brain. It is essential to further refine the OptoXRs based on structural analysis and Gα selectivity studies. Such efforts are crucial for elucidating the functions of GPCRs, providing deeper insights into their roles in specific brain regions and cell-specific events.

## Data Availability

All data reported in this study are available from the corresponding authors upon request.
