# Supplementary figures and images for: Optogenetic Control of Dopamine Receptor 2 Reveals a Novel Aspect of Dopaminergic Neurotransmission in Motor Function
Source: J Neurosci. 2024 Nov 19;45(1):e1473242024. doi: 10.1523/JNEUROSCI.1473-24.2024 (PMC11694400; doi:10.1523/JNEUROSCI.1473-24.2024)

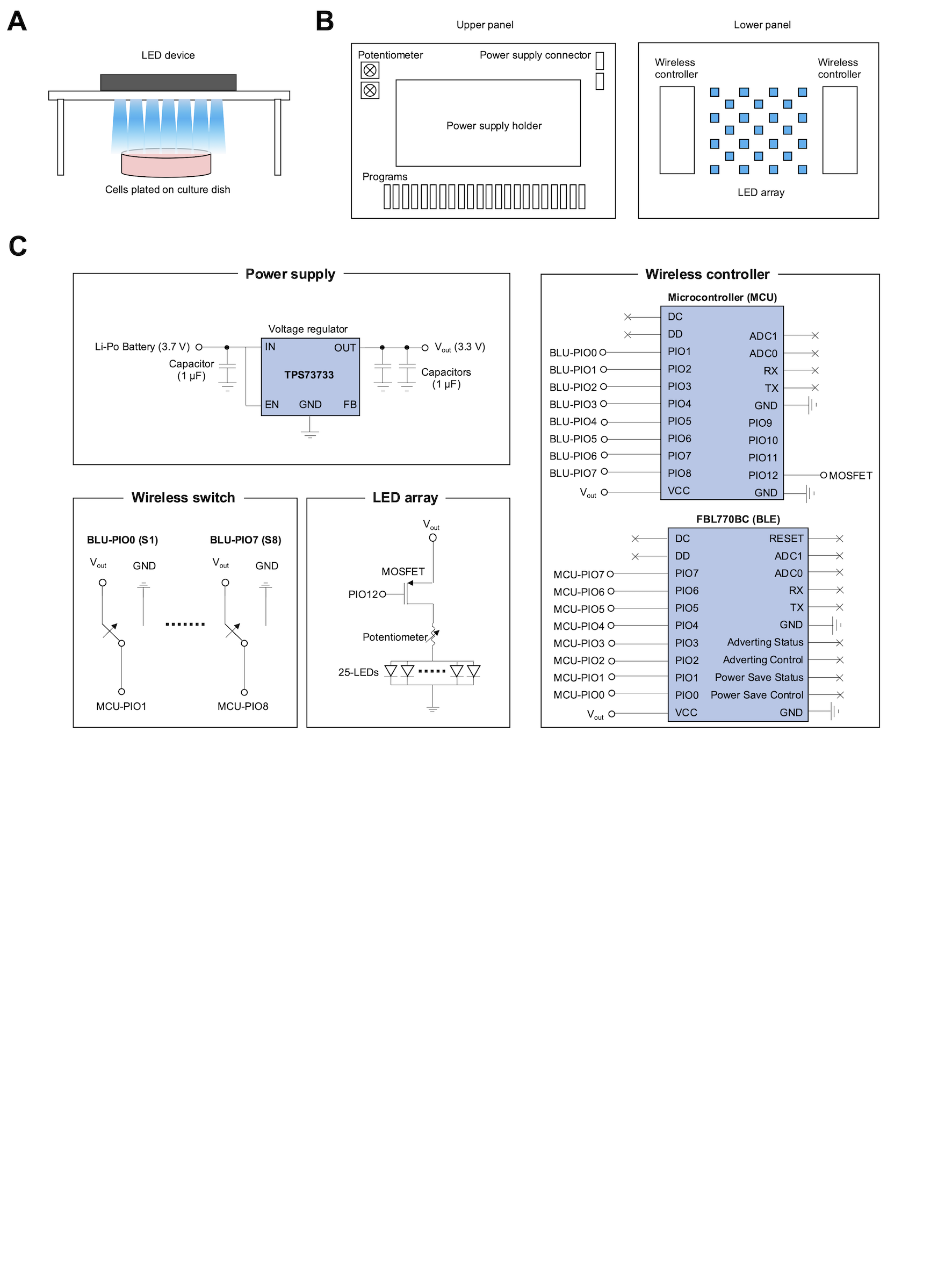

Supplement: Figure 2-1 — Development of the customized LED device. A, Design of the LED device for illuminating the cells expressing OptDRD2 from the top side of a culture dish. B, The LED array and wireless controllers are located in the lower panel of the device and the upper panel contains potentiometers, power supply connector and holder, and program channels. C, Workflows of the power supply, wireless switch, LED array, and wireless controller. Download Figure 2-1, TIF file. [file jneuro-45-e1473242024-s001.tif]

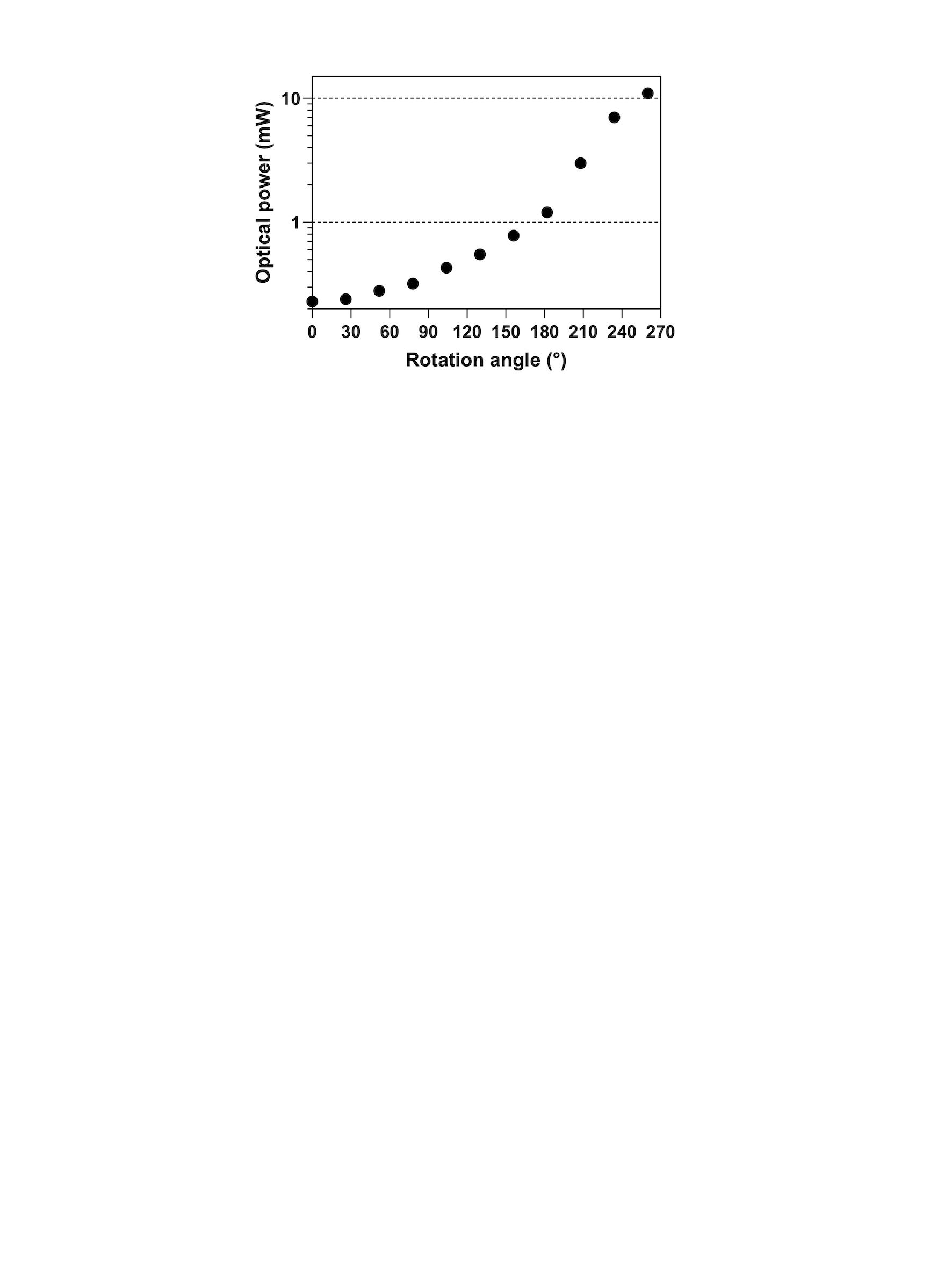

Supplement: Figure 2-2 — Optic power of the LED device according to rotation angle of the potentiometer. Download Figure 2-2, TIF file. [file jneuro-45-e1473242024-s002.tif]

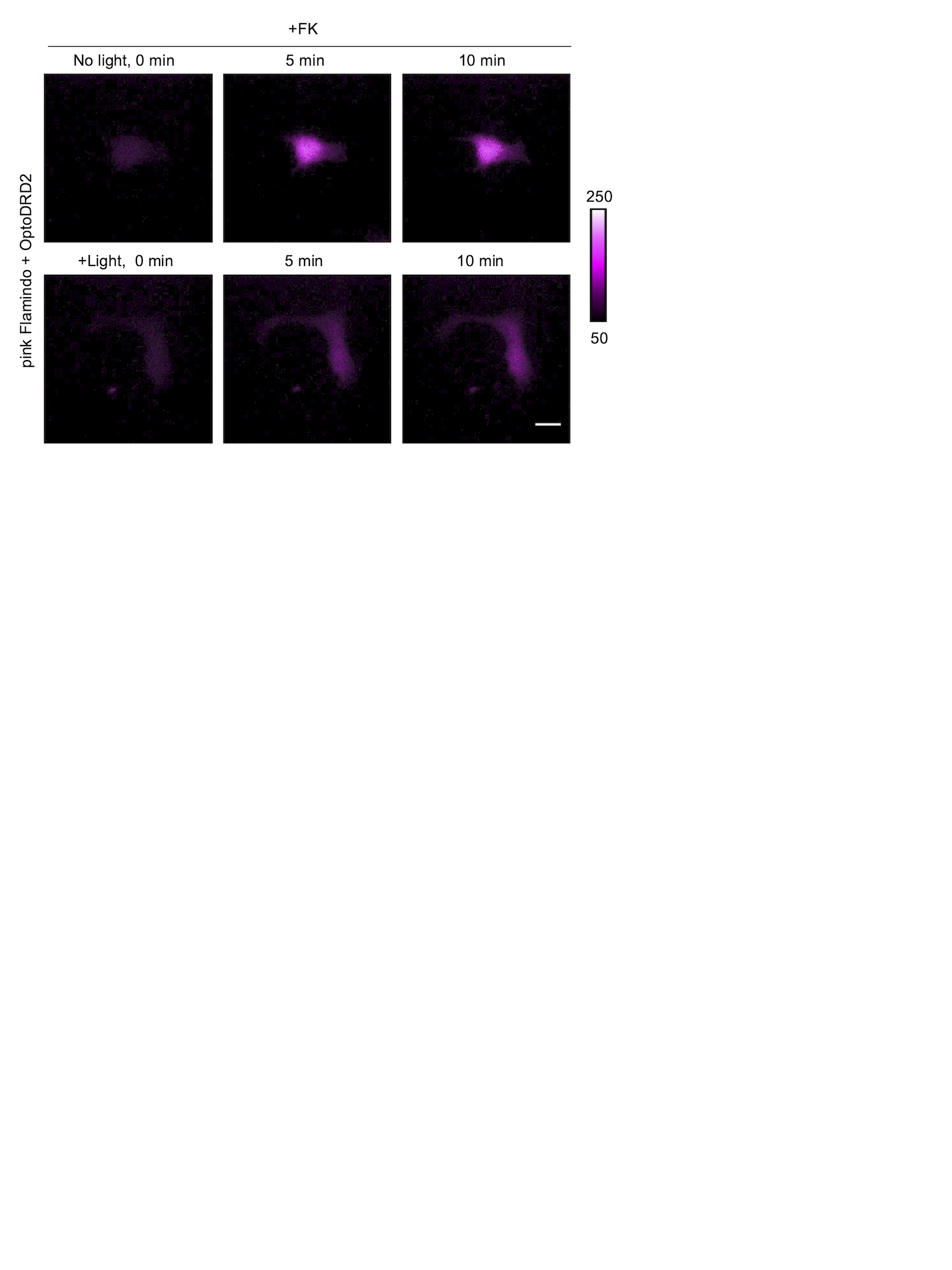

Supplement: Figure 2-3 — Visualization of the cAMP levels after the activation of OptoDRD1 and OptoDRD2. Representative images of the pink Flamindo (pF) in the cells expressing OptoDRD2 without or with illumination, under the treatment of 10 μM forskolin. Scale bar, 20 μm. Download Figure 2-3, TIF file. [file jneuro-45-e1473242024-s003.tif]

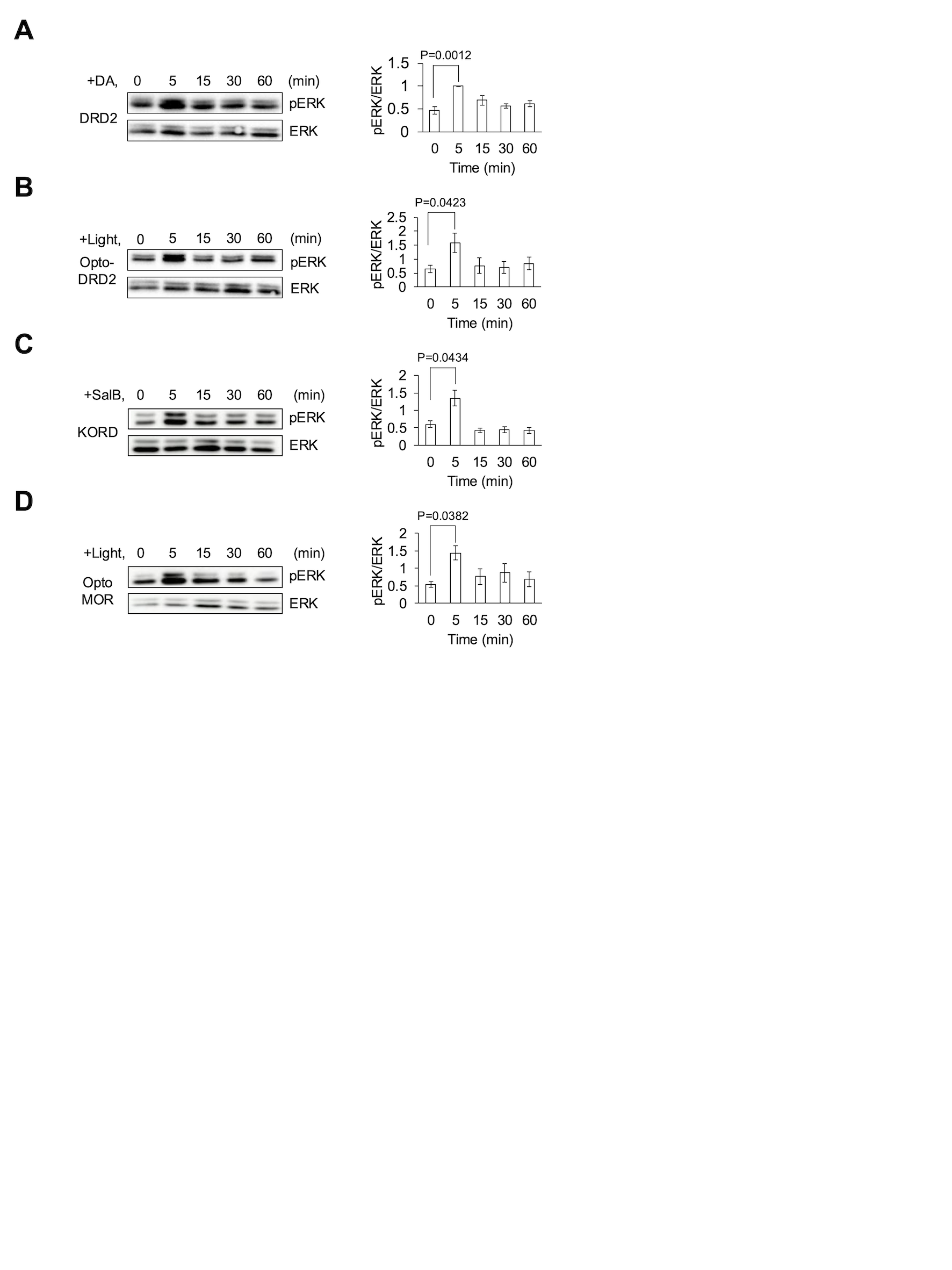

Supplement: Figure 3-1 — Time course of ERK phosphorylation of Gαi-type GPCRs upon activation. Time courses of ERK phosphorylation in the cells expressing DRD2 (n = 3) (A), OptoDRD2 (n = 5) (B), KORD (n = 4) (C) and OptoMOR (n = 3) (D) after light illumination or ligand treatment (10 μM dopamine for DRD2, 10 μM SalB for KORD). The graph shows the normalized ratio of phosphorylated ERK to total ERK levels. Data are shown as mean ± s.e.m. F = 8.123, dfn = 4, dfd = 10; p = 0.0012 (0 min vs. 5 min); p = 0.1485 (0 min vs. 15 min); p = 0.7331 (0 min vs. 30 min); p = 0.4279 (0 min vs. 60 min); (A, DRD2); F = 2.498, dfn = 4, dfd = 20; p = 0.0423 (0 min vs. 5 min); p = 0.9870 (0 min vs. 15 min); p = 0.9995 (0 min vs. 30 min); p = 0.9299 (0 min vs. 60 min); (B, OptoDRD2); F = 5.419, dfn = 4, dfd = 15; p = 0.0434 (0 min vs. 5 min); p = 0.7735 (0 min vs. 15 min); p = 0.7960 (0 min vs. 30 min); p = 0.5937 (0 min vs. 60 min); (C, KORD); F = 2.767, dfn = 4, dfd = 10; p = 0.0382 (0 min vs. 5 min); p = 0.8553 (0 min vs. 15 min); p = 0.6348 (0 min vs. 30 min); p = 0.9671 (0 min vs. 60 min); (D, OptoMOR). (one-way ANOVA followed by Dunnett’s multiple comparison test). Download Figure 3-1, TIF file. [file jneuro-45-e1473242024-s004.tif]

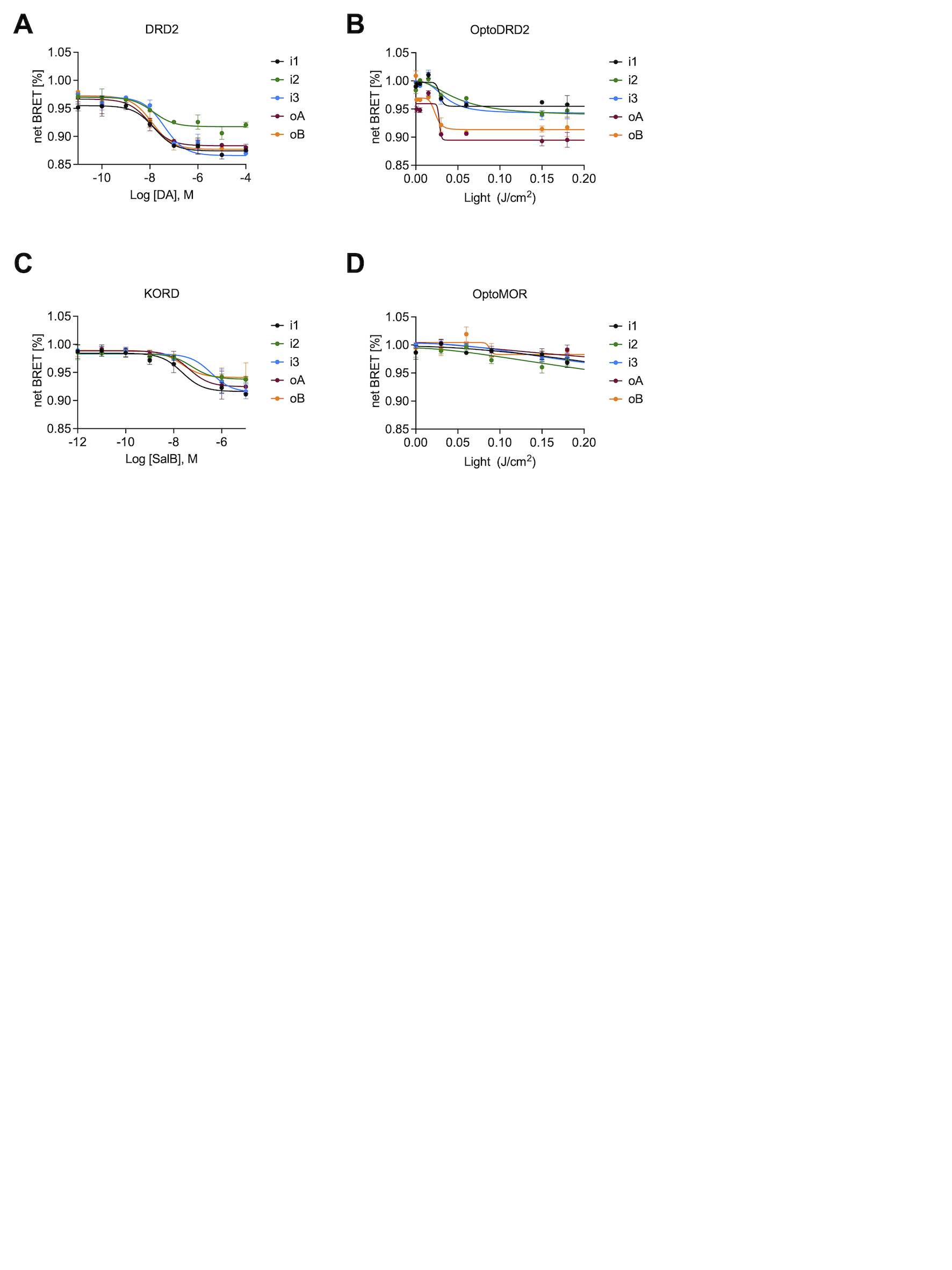

Supplement: Figure 3-2 — Different Gα subtype coupling profiles of Gαi/o type GPCRs. Dose-response curves of Gαi/o subtypes (i1, i2, i3, oA, oB) for DRD2 (A), OptoDRD2 (B), KORD (C), and OptoMOR (D) in response to light stimulation or drug treatment (dopamine for DRD2, SalB for KORD). Data are shown as mean ± s.e.m. (n = 4), n is the number of wells and each well contains 4 × 10^4^ cells. Download Figure 3-2, TIF file. [file jneuro-45-e1473242024-s005.tif]

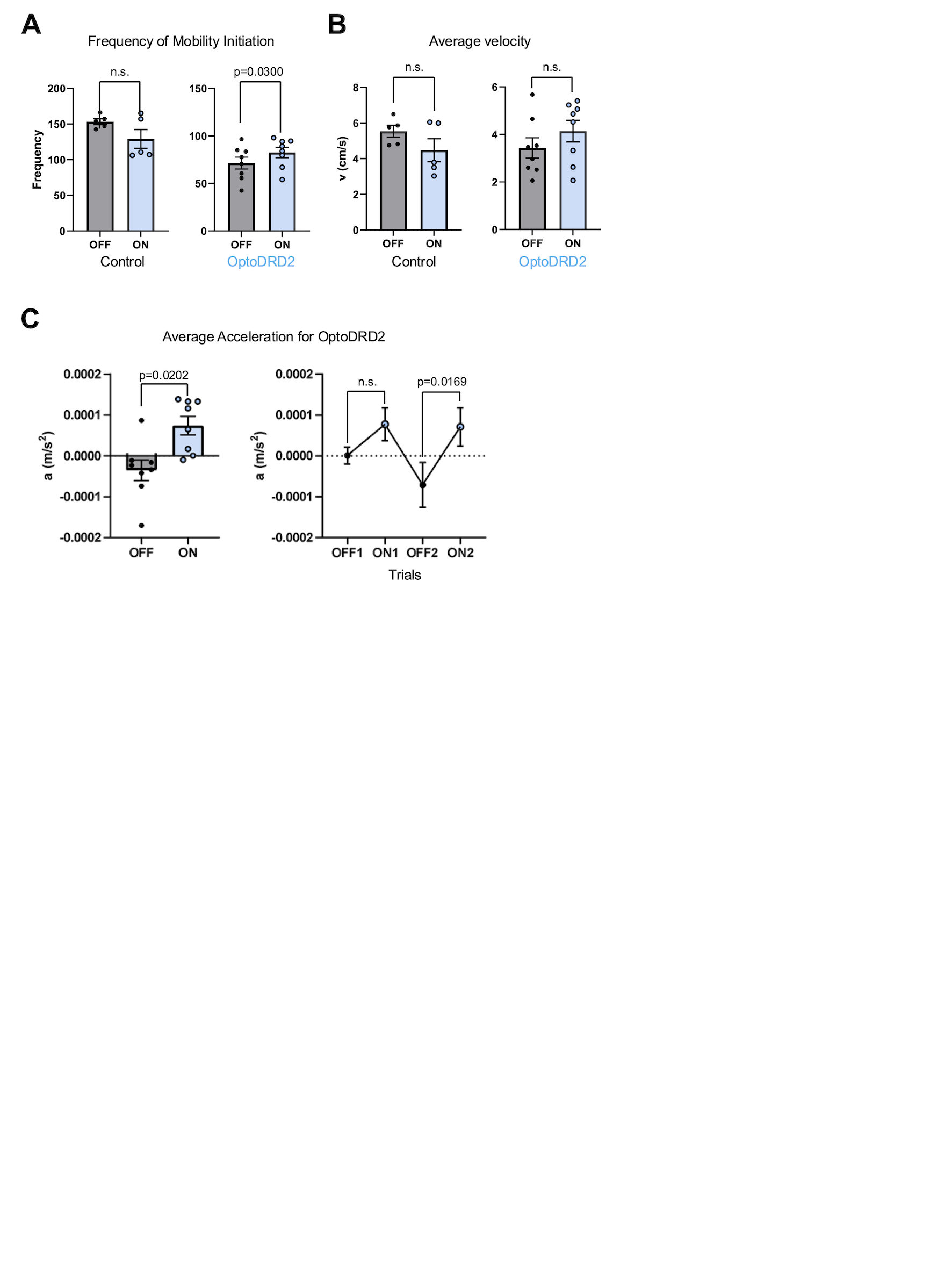

Supplement: Figure 7-1 — Optogenetic stimulation of CamKII(+) DRD2 neurons in LGP region expressing OptoDRD2 induces increased movement vigor. A, Frequency of mobility during ‘on’ and ‘off’ session, for control (n = 5) (left), OptoDRD2 (n = 8) (right). Data are shown as means ± s.e.m. pcontrol = 0.1351, tcontrol = 1.868, dfcontrol = 4; pOptoDRD2 = 0.0300, tOptoDRD2 = 2.714, dfOptoDRD2 = 7. (two-tailed paired t-test). B, Average velocity throughout the trial for control (left), OptoDRD2 (right). Data are shown as means ± s.e.m. pcontrol = 0.0677, tcontrol = 2.486, dfcontrol = 4; pOptoDRD2 = 0.1528, tOptoDRD2 = 1.604, dfOptoDRD2 = 7. (two-tailed paired t-test). C, Overall acceleration during on and off trials of OptoDRD2 animals (left), and by individual period (right) (n = 8). Data are shown as means ± s.e.m. p = 0.0202, t = 2.991, df = 7 (Overall acceleration); p = 0.0788, t = 2.057, df = 7 (Trial 1); p = 0.0169, t = 3.117, df = 7 (Trial 2). (two-tailed paired t-test). Download Figure 7-1, TIF file. [file jneuro-45-e1473242024-s006.tif]
